# Structural insights of the elongation factor EF-Tu complexes in protein translation of *Mycobacterium tuberculosis*

**DOI:** 10.1038/s42003-022-04019-y

**Published:** 2022-10-03

**Authors:** Bowen Zhan, Yanqing Gao, Wenqing Gao, Ye Li, Zhengyang Li, Qi Qi, Xin Lan, Hongbo Shen, Jianhua Gan, Guoping Zhao, Jixi Li

**Affiliations:** 1 State Key Laboratory of Genetic Engineering, School of Life Sciences, Shanghai Engineering Research Center of Industrial Microorganisms, Fudan University, 200438 Shanghai, China; 2 Shanghai Clinical Research Center for Infectious Disease (tuberculosis), Shanghai Pulmonary Hospital, Tongji University School of Medicine, 200433 Shanghai, China; 3 Key Laboratory of Synthetic Biology, CAS Center for Excellence in Molecular Plant Sciences, Shanghai Institute of Plant Physiology and Ecology, Chinese Academy of Sciences, 200031 Shanghai, China; 4 Shanghai Key Laboratory of Infectious Diseases and Biosafety Emergency Response, National Medical Center for Infectious Diseases, Huashan Hospital, Fudan University, 200040 Shanghai, China

**Keywords:** X-ray crystallography, Pathogens

## Abstract

Tuberculosis (TB) caused by *Mycobacterium tuberculosis* (Mtb) is the second-deadliest infectious disease worldwide. Emerging evidence shows that the elongation factor EF-Tu could be an excellent target for treating Mtb infection. Here, we report the crystal structures of Mtb EF-Tu•EF-Ts and EF-Tu•GDP complexes, showing the molecular basis of EF-Tu’s representative recycling and inactive forms in protein translation. Mtb EF-Tu binds with EF-Ts at a 1:1 ratio in solution and crystal packing. Mutation and SAXS analysis show that EF-Ts residues Arg13, Asn82, and His149 are indispensable for the EF-Tu/EF-Ts complex formation. The GDP binding pocket of EF-Tu dramatically changes conformations upon binding with EF-Ts, sharing a similar GDP-exchange mechanism in *E. coli* and *T. ther*. Also, the FDA-approved drug Osimertinib inhibits the growth of *M. smegmatis*, H37Ra, and *M. bovis* BCG strains by directly binding with EF-Tu. Thus, our work reveals the structural basis of Mtb EF-Tu in polypeptide synthesis and may provide a promising candidate for TB treatment.

## Abstract (web summary)

Crystal structures of M. tuberculosis elongation factors EF-Tu and EF-Ts in complex and GDP-bound Ef-Tu reveal the molecular basis of EF-Tu’s representative recycling and inactive forms in protein translation.

## Introduction

Tuberculosis, caused by *Mycobacterium tuberculosis* (Mtb), is one of the deadliest infectious diseases and has posed a severe threat to human health for thousands of years, accounting for about one million-death worldwide each year^1^. There are currently four types of drugs that can partially inhibit the growth of Mtb^1^. Isoniazid and its derivatives represent one category inhibiting cell wall formation, disturbing the synthesis of mycolic acid by producing nitric oxide in vivo, and affecting the type II fatty acid synthase (FAS) system^2^. Pyrazinamide, a prodrug of the active form pyrazinoic acid, can inhibit the FAS I enzyme and disrupt membrane potential, therefore interfering with Mtb’s survival at an acidic site of infection^3^. The third category of medicine, such as quinolone, acts on inhibiting DNA replication. Various fluoroquinolone-based drugs have shown promising effects against the DNA gyrase enzyme and, in turn, are successful in combating multidrug-resistant tuberculosis (MDR-TB)^4^. The last one, including the third-line anti-TB drug linezolid, targets ribosomes to inhibit protein synthesis by binding to 23S rRNA in the catalytic site of the 50S ribosome^5,6^. However, Mtb exhibits latency during infection, making antibacterial drug development challenging. In addition, the increasing prevalence of drug-resistant Mtb has further accentuated the need for novel antimycobacterial drugs^7^.

The ribosome is the protein translation machine in cells, and the protein synthesis needs the help of many accessory proteins during the initiation, elongation, and recycling processes^8,9^. The most common EFs in prokaryotes include EF-G, EF-Ts, and EF-Tu^10^. Although EFs in bacteria are homologous, they possess distinct structures and exert variant functions^11,12^. EF-Tu, one member of the GTPase family and the most abundant protein in bacteria, is responsible for catalyzing the binding of an aminoacyl-tRNA (aa-tRNA) to the ribosome by hydrolyzing GTP to GDP^13–15^. This process provides amino acids to elongate the newly synthesized protein via transferring the correct amino acid-binding in the A-site according to the codon-anticodon pairing. Upon binding with GTP, EF-Tu becomes an active form to bind with aa-tRNAs; then, EF-Tu-GTP recognizes the aminoacyl bond and has a high affinity for all aa-tRNAs by binding to the receptor motif^16^. Next, EF-Tu delivers aa-tRNAs to the mRNA-programmed ribosome, where the mRNA codon in the ribosomal A-site is recognized by aa-tRNA in a ternary complex with EF-Tu and GTP^17^. Also, the GTP hydrolysis by EF-Tu is activated by the cognate codon-anticodon interaction^18^. The additional rounds of ternary complex formation can occur on the ribosome during proofreading, to increase the rate and fidelity of aa-tRNA selection at the expense of GTP hydrolysis^19^. EF-Tu then hydrolyzes GTP to GDP, resulting in the EF-Tu•GDP complex having a low affinity to the ribosome, therefore releasing from the ribosome^20^. To be reactivated from the inactive form (EF-Tu•GDP) to the active form, EF-Tu needs to bind with EF-Ts (elongation factor thermo-stable), a GTPase-activating protein (GAP), in turn releasing the active EF-Tu•GTP^12^.

Many prokaryotic species encode two gene copies of EF-Tu, named *tufA* and *tufB*, which share high amino acid sequence similarity^21^. Reports showed that about two-thirds of EF-Tu was expressed from the *tufA* gene, and the other was expressed from the *tufB* gene in *E. coli* and *Salmonella typhimurium*^21,22^. Unlike other species, there is only one gene encoding EF-Tu in Mtb. Emerging evidence showed that EF-Tu could be an excellent target for treating infection from bacteria^23,24^. Currently, four compounds, including GE2270A, enactloxin lla, kirromycin, and pulvomycin, are used for targeting EF-Tu and inhibiting its activity^23,25,26^. The compounds inhibit either the dissociation between EF-Tu•GDP and ribosome, or the ternary complex formation. However, the compounds do not work well for Mtb^25,26^. Most importantly, although the homologic structure of *E. coli* has been reported^27^, the absence of structural information on Mtb EF-Tu and EF-Ts dampens the discovery of pharmacological intervention for treating Mtb.

Here, we report the crystal structures of Mtb EF-Tu•EF-Ts and EF-Tu•GDP complexes, elucidating the molecular basis of the representative recycling or inactive form of EF-Tu in protein translation in Mycobacterium, similar to reported in *E. coli* and *T. ther*. The GDP-binding pocket residues of EF-Tu dramatically changed conformations upon binding with EF-Ts, illustrating how EF-Ts reactivate the inactivated EF-Tu. Also, we identified that Osimertinib could inhibit different mycobacterium strains by direct targeting EF-Tu.

## Results

### Crystallization and structure determination of the Mtb EF-Tu•EF-Ts complex

**Fig. 1:**
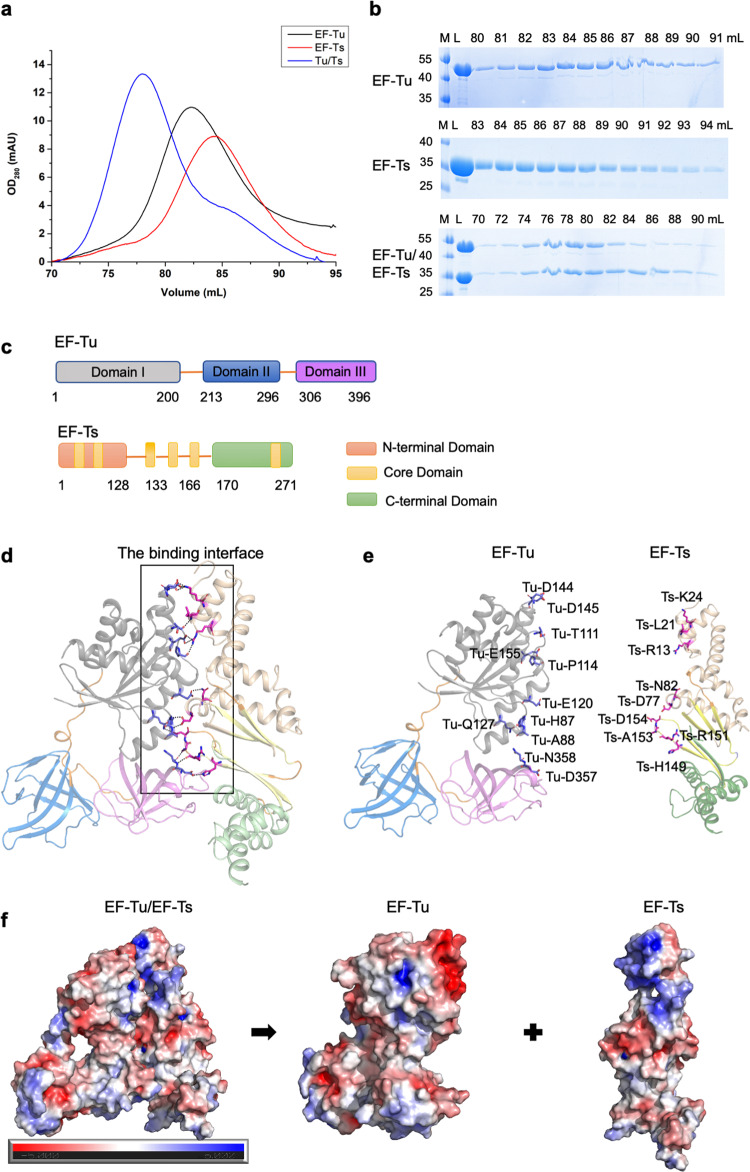
Crystal structure of Mtb EF-Tu•EF-Ts complex. **a** The size-exclusion chromatography (SEC) profiles of Mtb EF-Tu, EF-Ts, and EF-Tu•EF-Ts complex on a Superdex200 16/600 column. **b** The SDS-PAGE results of EF-Tu, EF-Ts, and EF-Tu•EF-Ts complex, corresponding with (**a**). M protein marker, L protein loading sample. **c** Schematic domains of *Mtb* EF-Tu and EF-Ts. EF-Tu consisted of three domains, including Domain I (gray), Domain II (blue), and Domain III (purple). EF-Ts consisted of three domains, including the N-terminal domain (wheat), the C-terminal domain (green), and the core domain in the middle (yellow). **d** A ribbon representation of Mtb EF-Tu•EF-Ts complex structure, colored with that in (**c**). EF-Tu and EF-Ts formed a 1:1 symmetric complex. The binding interface was labeled with a black rectangle for key residues depicted with stick models, and the cartoon is shown with a 50% transparency to highlight the key residues. **e** A ribbon representation of the detailed binding interface between EF-Tu and EF-Ts. The involved key residues were labeled and shown as sticks. **f** The electrostatic potential surfaces of Mtb EF-Tu•EF-Ts complex (left), EF-Tu (middle), and EF-Ts (right). Red denoted negative potential, and blue denoted positive potential.

To reveal the molecular basis of the EF-Tu reactivating mechanism in Mtb, we investigated the crystal structure of the full-length EF-Tu and EF-Ts complex. We expressed and purified EF-Tu and EF-Ts in *Escherichia coli* cells. EF-Tu and EF-Ts formed a 1:1 complex in solution, evidenced by the fact that the complex came out at the elution peak of 76 mL on a Superdex200 16/600 GL column, which corresponds to a molecular mass of 71 kDa (Fig. 1a, b). Also, the EF-Tu or EF-Ts was eluted out at the peaks of 84 mL or 86 mL on the gel-filtration column, corresponding to a mass of 43 kDa and 30 kDa, respectively (Fig. 1a, b). The Mtb EF-Tu•EF-Ts crystals were obtained after several rounds of optimization, and the complex structure was solved at a resolution of 2.8 Å by molecular replacement method using the EF-Tu•GDP (PDB: 7VOK) and the AlphaFold-predicted EF-Ts (https://alphafold.ebi.ac.uk/entry/P9WNM1) as the searching models.

### The structure of Mtb EF-Tu•EF-Ts complex

The Mtb EF-Tu contains three domains, named Domain I (residues 1–200), II (residues 213–296), and III (residues 306–396) (Fig. 1c and Supplementary Fig. 1a). Domain I, also called the GTP-binding domain or Ras-like domain, hydrolyzes GTP to GDP in an Mg^2+^-dependent manner. Domains II and III are oligonucleotide-binding domains, which bind to both charged tRNA and EF-Ts in *T. thermophilus*^28^. The Mtb EF-Tu structure showed three domains forming a flattened triangular shape with a hole in the middle, arranged like other homolog structures (Fig. 1d and Supplementary Fig. 1a). Moreover, Domains I and III were closer than Domain I and II, making face-to-face contact and resulting the side-chain interactions. Also, the flattened triangular shape permits a high degree of inter-domain flexibility, which benefits EF-Tu to bind with kinds of substrates in the polypeptide synthesis^29,30^. The EF-Ts comprises 13 α-helices and 6 β-strands, containing three domains, the N-terminal domain, the core domain, and the C-terminal domain (Fig. 1c and Supplementary Fig. 1b). The core domain comprises two subdomains, denoted as subdomain N (residues 61–65, 68–73, and 133–139) and subdomain C (residues 144–149, 157–166, and 259–267), which is composed of 6 β-strands surrounding by the α-helices (Supplementary Fig. 1b).

The Mtb EF-Tu•EF-Ts complex contains one EF-Tu and one EF-Ts per asymmetric unit (Fig. 1d), different from that in *E. coli* and *T. thermophilus* that is composed of two EF-Tu and two EF-Ts molecules^27,28,31^. In *E. coli*, the amino-terminal region of EF-Ts interacts with the nucleotide-binding Domain I of EF-Tu, and the other half binds to Domain III. In addition, residues K51, G54, D80, F81, I125, and G126 of *E. coli* EF-Ts are vital for the interaction with EF-Tu; however, the structure and sequence alignment shows that only D80 (corresponding to D77 in *Mtb* EF-Ts) is the conserved active site^27^. In *T. thermophilus*, EF-Ts is a dimer, in which both subunits contribute to the bipartite interface. Also, the conserved TDFV sequence of EF-Ts contacts with EF-Tu Domain I in *E. coli*, *T. thermophilus*, and *B.mitochondrial*^32^. Similar to homologs in other species, the Mtb EF-Tu bound with the N-terminal domain and the core domain of EF-Ts through Domains I and III (Fig. 1d, e). We chose the pair of amino acids with a relative distance within 3 Å as interface residues. Twenty residues were involved in the interface with hydrogen bonds or salt bridges (Fig. 1d, e). Also, the electrostatic potential surfaces analysis showed that the EF-Tu•EF-Ts complex was full of negative potential surfaces (Fig. 1f). Notably, the binding interface of EF-Tu was full of negative potential, while that in EF-Ts was positive potential, indicating the charge-charge interaction promoting the complex formation, which is critical for protein stacking^33,34^.

### Mtb EF-Ts exhibits a high binding affinity to EF-Tu

**Fig. 2:**
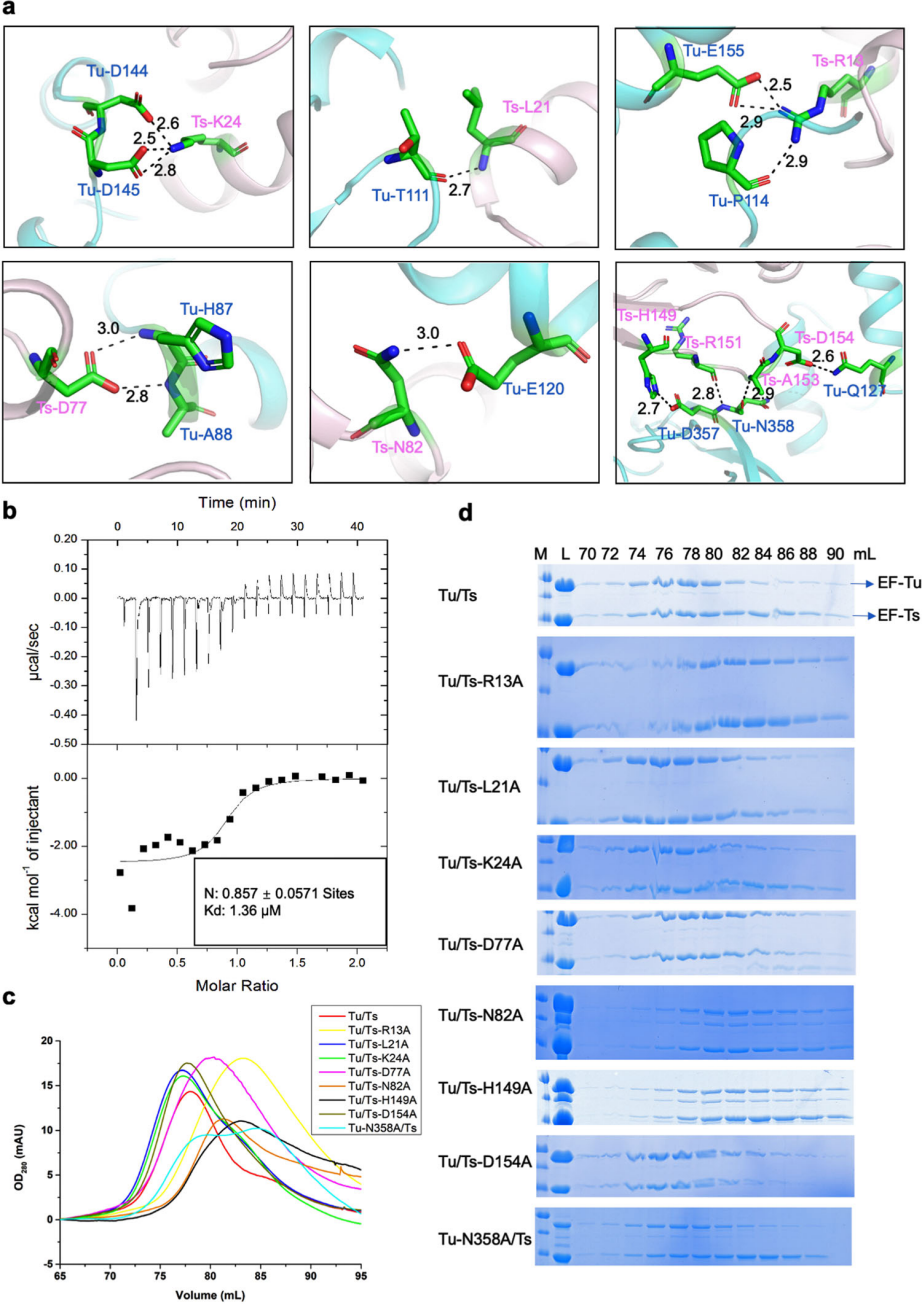
Mtb EF-Tu exhibits a high binding affinity with EF-Ts. **a** Ribbon representations of crucial residues in the interface between EF-Tu (cyan) and EF-Ts (light pink). The residues were labeled and shown as sticks. The hydrogen bonds among interacted residues are shown in black dashed lines. **b** The isothermal titration calorimetry (ITC) result showed that the Kd value between EF-Tu and EF-Ts was 1.36 µM. The binding molar ratio was around 1:1. **c** The SEC results of the EF-Tu•EF-Ts complex and different mutants. The mutants Tu/Ts-R13, Tu/Ts-N82, and Tu/Ts-H149 dramatically changed their solution status. Tu/Ts is abbreviated for EF-Tu/EF-Ts. **d** The SDS-PAGE result of the EF-Tu•EF-Ts complex and different mutants, corresponding to (**c**). M protein marker, L protein loading sample.

The interface-involved residues of EF-Tu were located in Domain I and III, while the related residues of EF-Ts were mainly located in the N-terminal domain and the core domain (Fig. 1d). Briefly, residue K24 on the α2 helix of EF-Ts formed salt bridges with residues D144 and D145 of EF-Tu, and the K24 side chain formed a hydrogen bond with D145. Residue R13 on the α1 helix of EF-Ts formed hydrogen bonds with P114 and E155 of EF-Tu, respectively. Moreover, residue D77 of EF-Ts formed a hydrogen bond with residue H87, and a salt bridge with A88 of EF-Tu. Residue N82 of EF-Ts formed a hydrogen bond with the side chain of EF-Tu E120. In addition, the N atom on the side chain of EF-Ts-H149 formed a salt bridge with the O atom of the D357 side chain of EF-Tu. Also, the side chain of EF-Ts R151 formed a hydrogen bond with N358 of EF-Tu, and D154 formed a salt bridge with EF-Tu Q127 (Fig. 2a). Next, we investigated the interaction between EF-Tu and EF-Ts using the isothermal titration calorimetry (ITC) method. The result showed that EF-Tu bound with EF-Ts at a 1:1 ratio with a Kd value around 1.36 µM (Fig. 2b), different from the Kd values for EF-Tu and EF-Ts from *E. coli* and *Bovine mitochondria* (2 nM and 5.5 nM, respectively)^35,36^. Reports showed that the equilibrium dissociation constants would change among different species despite the high sequence similarity^37^. Furthermore, Mtb EF-Tu shared 74% and 71% similarities in amino acid sequence with that in *E. coli* and *T. thermophilus*, and the interfaced-related residues were highly conserved (Supplementary Fig. 2). However, EF-Ts’ amino acid sequence similarity between Mtb and *E. coli*, or *T. thermophilus* was 39% or less, except that residues R13, K24, and D77 in Mtb were conserved (Supplementary Fig. 3).

To identify the critical residues for the EF-Tu/EF-Ts complex’s interaction, we mutated seven residues of EF-Ts into alanine, including R13, L21, K24, D77, N82, H149, D154, and EF-Tu N358. When EF-Tu was co-purified with the wild-type or mutant of EF-Ts on the size exclusion chromatography, it clearly showed that mutants R13A, N82A, and H149A dramatically disrupted the interaction profiles with EF-Tu, indicating the three residues are indispensable for the EF-Tu/EF-Ts formation (Fig. 2c, d).

### EF-Ts exhibits a diverse binding conformation among different species

**Fig. 3:**
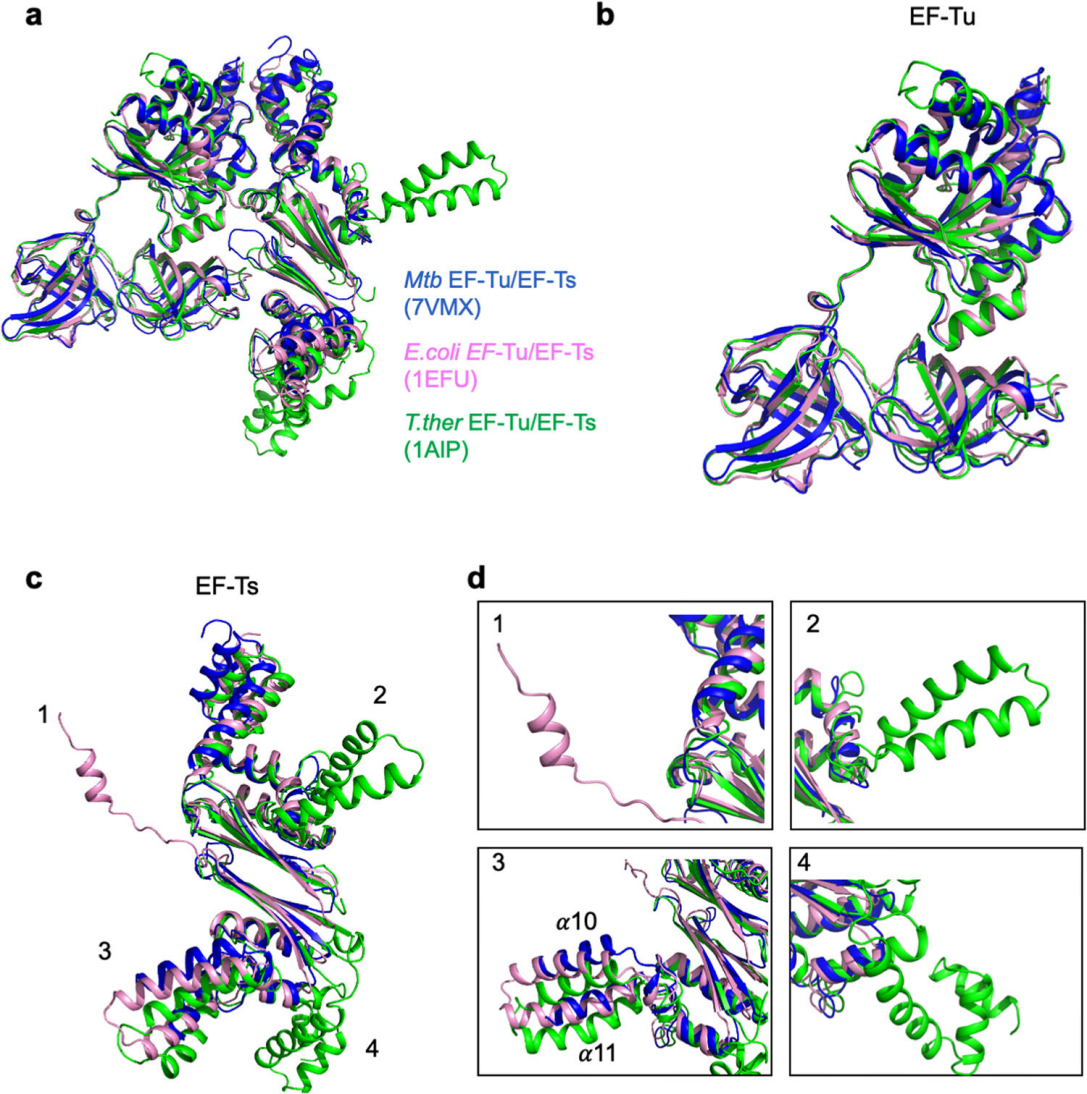
EF-Ts exhibits a diverse binding conformation among different species. **a** Superimposition of EF-Tu•EF-Ts complex structures among Mtb (marine, PDB: 7VMX), *E. coli* (light pink, PDB: 1EFU), and *T. thermophilus* (green, PDB: 1AIP). The RMSD values between Mtb and *E. coli* or *T. thermophilus* were 1.214 Å or 1.309 Å, respectively. **b** Superimposition of EF-Tu structures among Mtb, *E. coli*, and *T. thermophilus*. **c** Superimposition of EF-Ts structures among Mtb, *E. coli*, and *T. thermophilus*. The numbers denoted significant differences among the three structures. **d** The detailed difference of EF-Ts structures among three species, according to (**c**). ‘1’, the extra helix of *E. coli* EF-Ts for binding with EF-Tu Domain I. ‘2’ and ‘4’, the helices in the *T. thermophilus* EF-Ts, absent in Mtb and *E. coli*, which were not involved in binding with EF-Tu. ‘3’, the α10 and α11 helices that shifted about 4.7 Å or 6.0 Å within three species, respectively.

EF-Ts functions as a guanine nucleotide exchange factor and catalyzes the reaction of EF-Tu from the inactive form (GDP-bound) to the active form (GTP-bound)^38^. As EF-Ts shared low similarities among different species (Supplementary Fig. 3), and preferred to bind with and reactivate EF-Tu at different ratios between Mtb (1: 1, EF-Ts: EF-Tu) and *T. thermophilus*, (2:1, EF-Ts: EF-Tu)^28,31^, we compared the EF-Tu•EF-Ts complex structures using the ChimeraX and PymoL software. Superimposing the Mtb EF-Tu•EF-Ts complex with that of *E. coli* (PDB ID:1EFU) or *T. thermophilus* (PDB ID: 1AIP) revealed similar features of overall folds, which showed high similarities among the Cα atoms with RMSD values of 1.214 Å or 1.309 Å, respectively (Fig. 3a). In brief, the structural differences existed mainly in EF-Ts, while EF-Tu had similar architecture (Fig. 3a, b). Different from the Mtb EF-Ts, the EF-Ts of *E. coli* was composed of 13 α-helices and 6 β-strands, which assembled in four critical domains, the N-terminal domain (residues 1–54), the core domain (residues 55–179), the dimerization domain (residues 180–228), and the C-terminal domain (residues 264–282)^27^. The C-terminal domain was stretched out to interact with Domain I in EF-Tu of *E. coli*, whereas it was absent in Mtb EF-Ts (Fig. 3c, d and Supplementary Fig. 3)^27,28^, implying a distinct binding conformation in different bacteria.

The second significant difference was that the *T. thermophilus* EF-Ts had one extra helix between the β1 and β2 strands. As the EF-Tu•EF-Ts complex is an asymmetrical heterotetramer in *T. thermophilus*, of which one EF-Tu interacts with two subunits of EF-Ts, forming a bipartite interface; the extra helix could explain why one EF-Tu molecule needed to be reactivated by two EF-Ts molecules in *T. thermophilus* (Fig. 3d). Mtb and *E. coli* EF-Ts is a monomer with a structural repeat that mimics the dimeric interface in *T. thermophilus* EF-Ts^28^. Also, the helices, α10 and α11, of Mtb EF-Ts dramatically shifted with distances around 4.7 Å and 6.0 Å to that of *E. coli* or *T. thermophilus* (Fig. 3d).

### The Mtb EF-Tu•GDP complex structure

**Fig. 4:**
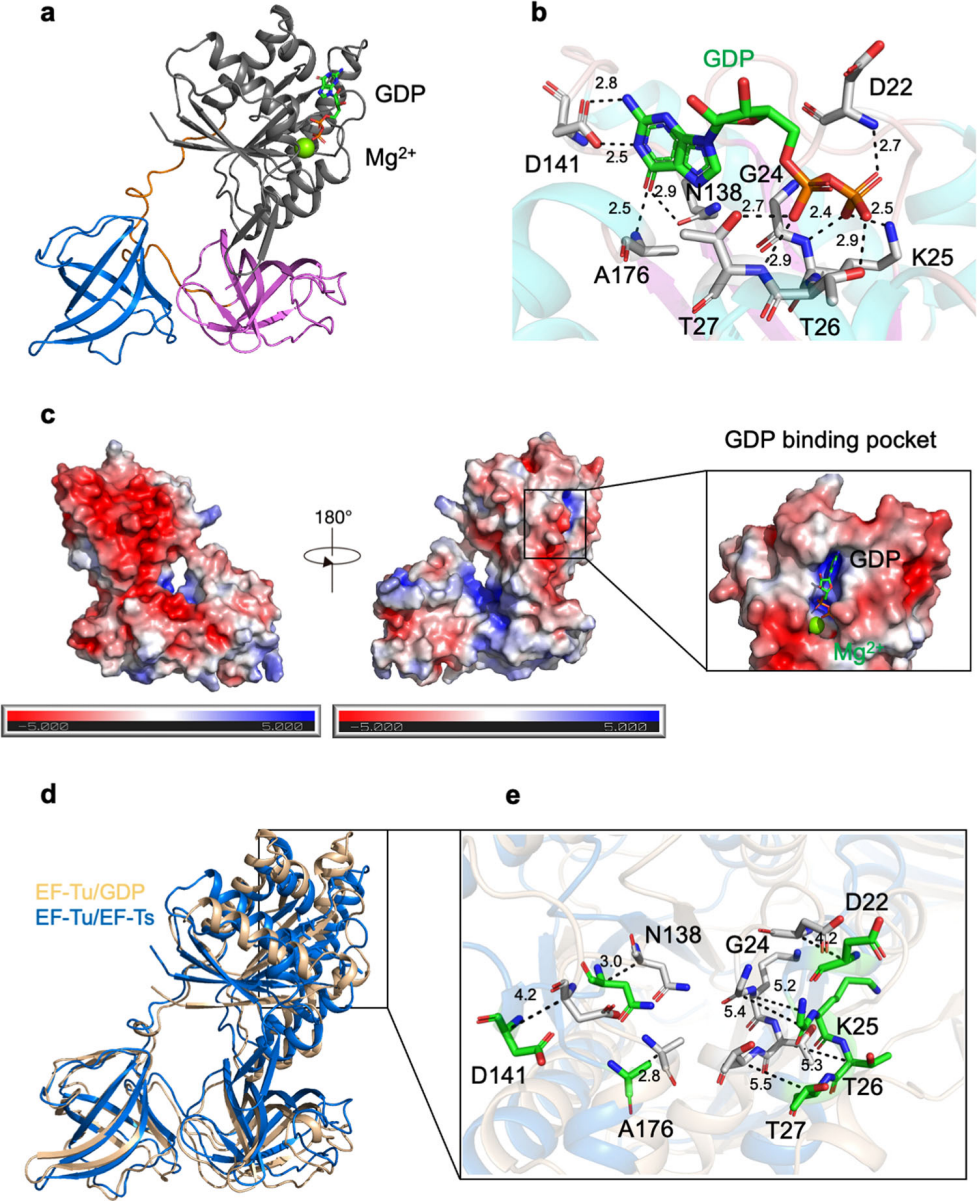
Critical residues in the GDP-binding pocket. **a** A ribbon representation of the EF-Tu•GDP structure, which is colored as Fig. 1a. GDP and Mg^2+^ were located in Domain I. GDP is shown as sticks, and Mg^2+^ is shown as a green sphere. **b** Representation of GDP-binding sites in the EF-Tu•GDP complex. The broken black lines represented the hydrogen bonds formed between EF-Tu and GDP. The related residues were labeled and shown as sticks. **c** The electrostatic potential surface of the EF-Tu•GDP complex. Red indicated the negative potential, and blue indicated the positive potential. GDP is shown as sticks, and Mg^2+^ is shown as a green sphere. **d** Superimposition of Mtb EF-Tu between the EF-Tu•GDP (yellow) and EF-Tu•EF-Ts (marine) complexes. The RMSD value for the Cα alignment between the two structures was 0.711 Å. **e** The detailed shift of GDP-bound residues between the EF-Tu•GDP and EF-Tu•EF-Ts complexes. The broken black lines represented the shifting of labeled residues.

To elucidate the molecular basis of the inactive form of Mtb EF-Tu during the elongation cycle, we expressed and purified the full-length EF-Tu protein, and successfully obtained the EF-Tu•GDP complex structure at a resolution of 3.4 Å using the *E. coli* EF-Tu (PDB ID: 1EFC) as a searching model. The final structure contains four EF-Tu molecules per asymmetric unit (Table 1). Each EF-Tu molecule was bound with a GDP and an Mg^2+^ ion (Fig. 4a). Domain I of Mtb EF-Tu underwent a structural rearrangement of approximately 90° rotation to the other domains (Fig. 4a), similar to that in *E. coli* which occurred around switches 1 and 2 when GTP replaced GDP. This movement formed a binding site of aminoacyl-tRNA to interact with all three domains of EF-Tu. Domain II was composed of 84 residues from 213 to 296, with 6 antiparallel β-strands forming a β-barrel (Supplementary Fig. 1a), different from that in *E. coli* EF-Tu. Residues of Domain II took responsibility for discriminating between the charged and uncharged tRNA in the binding pocket. Moreover, Domain III of Mtb was composed of 91 residues from 306 to 396, with 6 antiparallel β-strands forming a β-barrel. In Mtb, Domains II and III were connected by a shorter peptide, but in *E. coli*, there was one more β-strand between the two domains^20^. Also, Domain II and III formed antiparallel β-barrels to regulate the activity of Domain I, increasing the affinity of GDP over GTP (Fig. 4)^38^.

**Table 1:** Data collection and refinement statistics.

	EF-Tu•EF-Ts	EF-Tu•GDP
PDB code	7VMX	7VOK
Data collection		
Space group	P 4_3_ 2_1_ 2	P 2_1_
Unit cell a, b, c (Å)	128.11, 128.11, 200.67	95.06, 75.18, 127.18
α, β, γ [°]	90, 90, 90	90, 93.77, 90
Resolution range	29.86–2.80 (2.90–2.80)*	29.95–3.40 (3.52–3.40)*
Total reflections	1,095,359 (110,789)	169,671 (17,398)
Unique reflections	41,658 (1339)	24,414 (663)
Multiplicity	26.3 (27.1)	6.9 (7.2)
Completeness (%)	98.9 (91.2)	94.9 (90.7)
* I*/*σ* (I)	5.0 (3.46)	7.9 (4.97)
Wilson B-factor	48.56	53.02
R_merge_	0.141 (0.535)	0.094 (0.317)
CC_1/2_	0.998	0.998
Refinement statistics		
Resolution	29.86–2.80 (2.88–2.80)	29.95–3.40 (3.58–3.40)
Reflections used in refinement	36,510 (1339)	20,005 (663)
Reflections used for R-free	1815 (64)	1017 (29)
R_work_/R_free_ (%)	23.16/26.77	24.43/30.16
Protein residues	619	1529
No. of atoms		
Number of non-hydrogen atoms	4717	11,912
Macromolecules	4706	11,796
Ligands or ions	7	116
Solvent	4	–
RMSD		
RMS (bonds)	0.015	0.008
RMS (angles)	2.20	1.43
Ramachandran favored (%)	92.14	94.63
Ramachandran allowed (%)	7.53	4.71
Ramachandran outliers (%)	0.33	0.66
B-factors		
Average B-factor	63.5	53.0
Macromolecules	63.49	53.38
Ligands	83.60	64.92
Solvent	36.56	–

*Values in parentheses are for the highest-resolution shell.

R_merge_ = ∑|Ii − <I> | /∑ |I| , where Ii is the intensity of an individual reflection, and I is the average intensity of that reflection.

R_work_ = ∑||F_o_| − |F_c_| |/ ∑|F_o_| , where F_o_ and F_c_ are the observed and calculated structure factors for reflections, respectively.

R_free_ was calculated as R_work_ using the 5% of reflections that were selected randomly and omitted from refinement.

### The GDP-binding sites are conserved among different species

The GDP molecule was bound with Domain I of EF-Tu, which consisted of five loops, named G-1 to G-5, similar to other nucleotide-binding proteins (Fig. 4b and Supplementary Fig. 2)^28,31,39^. G-1 was referred to as the P-loop due to the conserved amino acid sequence like GX4GK(ST) from residues G19 to K25, which connected the β1 strand and α1 helix responsible for phosphate binding. Also, residues K25, T26, and T27, located on the α1 helix, forming hydrogen bonds with the α- and β-phosphate of GDP. G-2 contained the second β-strand and its preceding loop, which was bound with the γ-phosphate of GTP described in other homologous structures. G-3 contained the conserved sequence of DX_4_G, residues from 83 to 86 in Mtb EF-Tu, which connected the β5 strand and α3 helix. Especially, residue D83 was bound with the Mg^2+^ ion through several water molecules as described before. G-4 contained β5 strand and its preceding loop, and had the conserved amino acids sequence of (N/T) (K/Q) XD, which was NKAD in Mtb EF-Tu spanned from residues 138 to 145 that formed a loop to connect β7 strand and α5 helix. Also, residues D141 and A176 formed hydrogen bonds with the guanine ring of GDP. The G-5 region was composed of β8 strand and α6 helix, which spanned from residues P170 to A176, and was far from the GDP and Mg^2+^ molecules (Fig. 4b).

The GDP molecule interacted with residues D22, G24, K25, T26, T27, N138, D141, and A176 of EF-Tu, which were conserved in Mtb, *E. coli*, and *T. thermophilus* (Fig. 4b and Supplementary Fig. 2). Residues D22 and G24 were located in the first loop, which connected the β1 strand and α1 helix. Also, D141 was located in the loop connecting the β7 strand and α5 helix. Moreover, analyzing the electrostatic potential surface showed that GDP was bound in the pocket full of positive charges (Fig. 4c).

When superimposing the EF-Tu molecules in the EF-Tu•GDP and EF-Tu•EF-Ts structures, it showed similar features of overall folds with an RMSD value of 0.711 Å (Fig. 4d). A significant difference existed in Domains I and III, responsible for the interaction with EF-Ts. The EF-Tu in the recycling complex (EF-Tu•EF-Ts) had a much more compact shape than that in the inactive form (EF-Tu•GDP) (Fig. 4d). Significantly, the residues involved in the GDP-binding pocket changed positions. D22, G24, K25, T26, T27, A176, and D141 of EF-Tu shifted from 2.8 Å to 5.5 Å upon conformational change (Fig. 4e), which could explain how EF-Ts help EF-Tu to dissociate with GDP and reactivate EF-Tu during the elongation cycle of protein translation. The pocket was no longer suitable for binding with GDP because of the translocate of GDP-binding residues after EF-Ts binding, similar to the report in *E. coli*, in which the movement of residues K136 and N138 of EF-Tu made them away from the nucleotide-binding site and relaxed the interactions within the base (Fig. 4e)^38^.

**Fig. 5:**
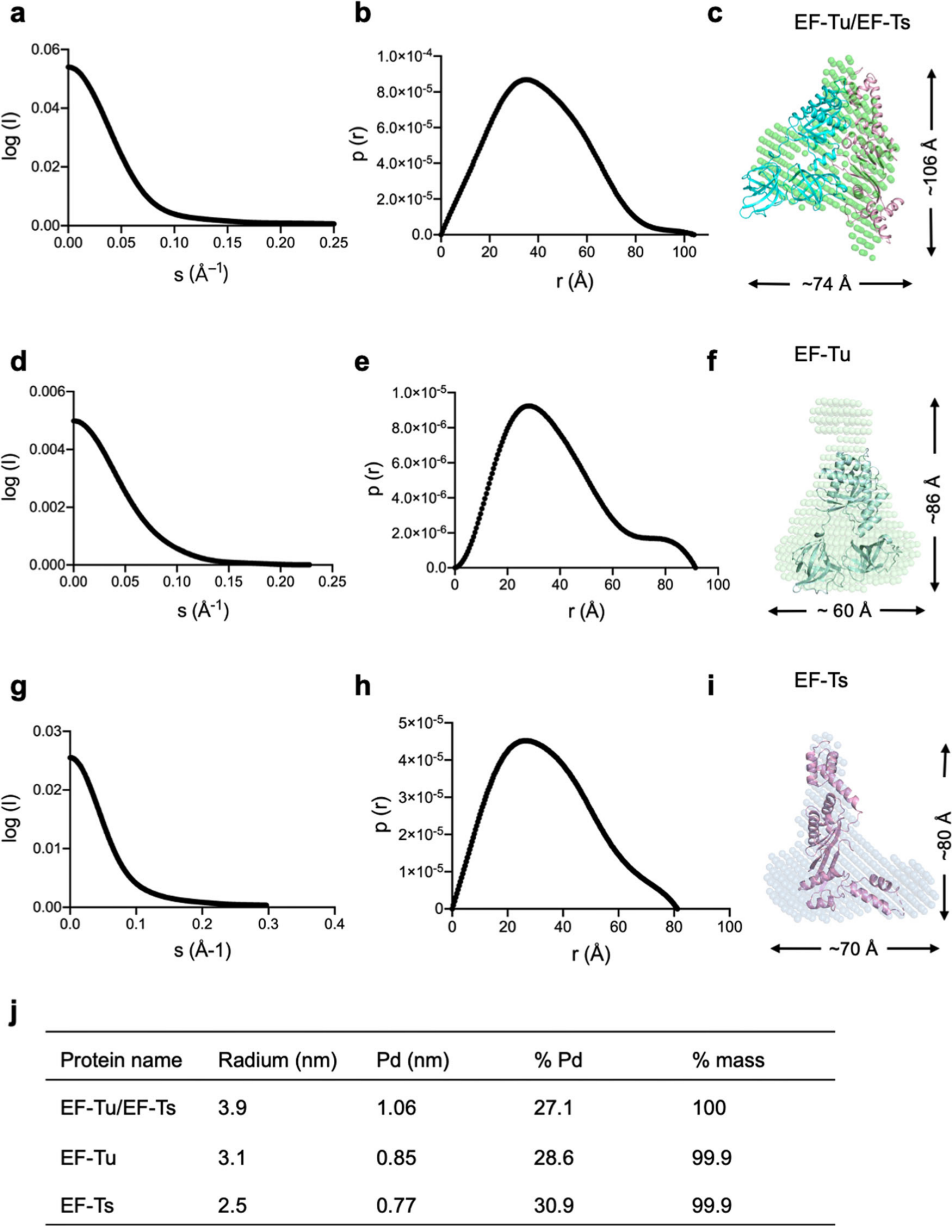
The SAXS models of the Mtb EF-Tu, EF-Ts, and EF-Tu•EF-Ts complex. **a** The experimental scattering curve of the Mtb EF-Tu•EF-Ts complex. **b** The distance distribution function curve of the EF-Tu•EF-Ts complex. **c** The crystal structure of the EF-Tu•EF-Ts complex was fitted into the ab initio envelope obtained from SAXS. **d** The experimental scattering curve of Mtb EF-Tu. **e** The distance distribution function curve of Mtb EF-Tu. **f** The crystal structure of EF-Tu was fitted into the ab initio envelope obtained from SAXS. **g** The experimental scattering curve of Mtb EF-Ts. **h** The distance distribution function curve of Mtb EF-Ts. **i** The crystal structure of EF-Ts was fitted into the ab initio envelope obtained from SAXS. **j** Dynamic light scattering (DLS) analysis of the Mtb EF-Tu, EF-Ts, and EF-Tu•EF-Ts complex. Particle polydispersity was defined in the following terms: polydispersity (Pd), % polydispersity (% Pd).

Furthermore, we investigated the solution status of EF-Tu, EF-Ts, and EF-Tu•EF-Ts complex by the small-angle X-ray scattering (SAXS) method (Fig. 5a–i). All the proteins behaved well in solution. The maximum dimension (Dmax) from distance distribution function p(r) for the EF-Tu, EF-Ts, and EF-Tu•EF-Ts complex were 86 Å, 80 Å, and 106 Å, respectively (Fig. 5b, e, h). When superimposed the crystal structures with the envelopes generated from SAXS data, EF-Ts and EF-Tu•EF-Ts complex showed high similarities, whereas EF-Tu had discrepancies resulting from a highly flexible loop between Domain I and Domain II (Fig. 5c, f, i).

### The FDA-approved drug Osimertinib binds with Mtb EF-Tu and inhibits *Mycobacterium* growth

**Fig. 6:**
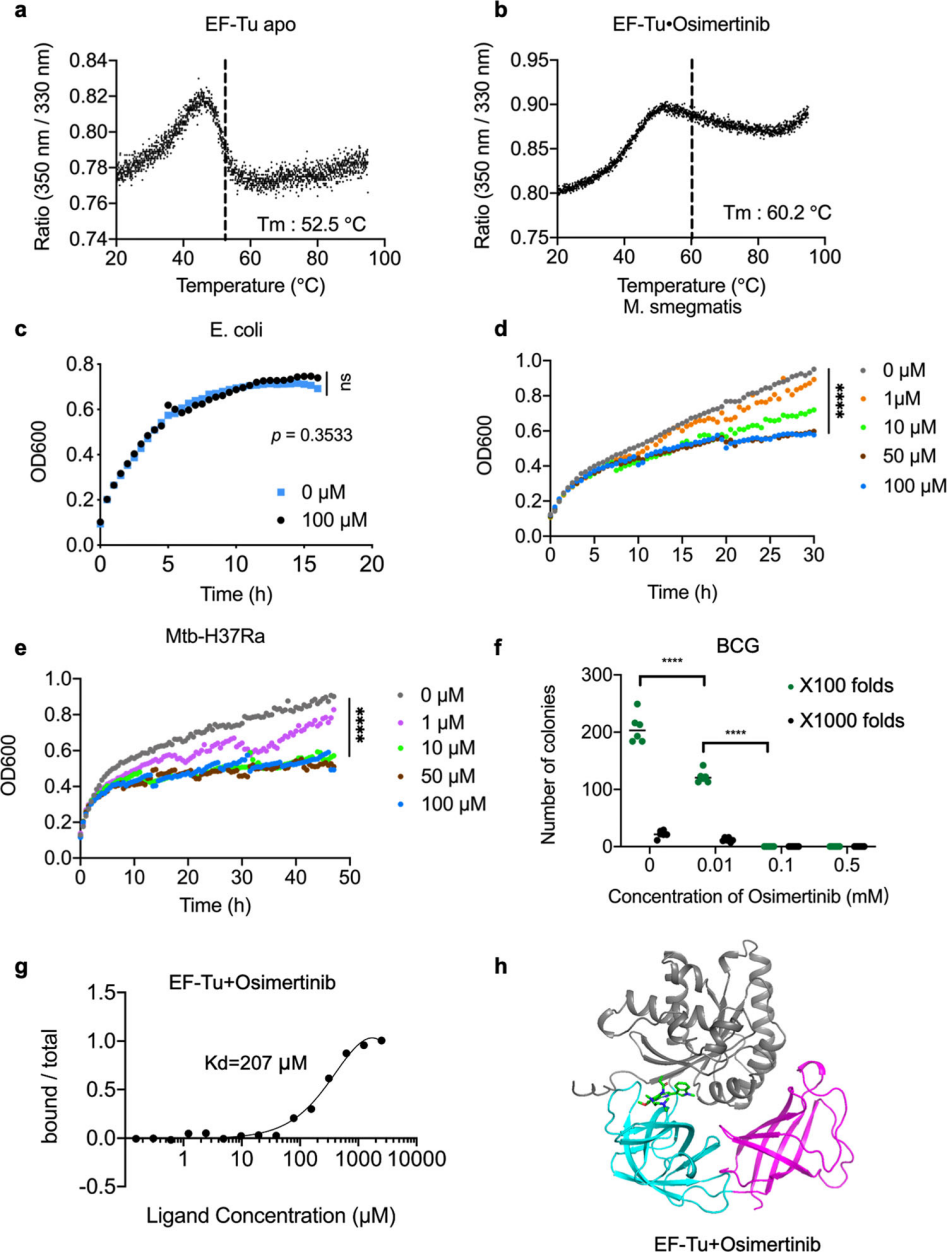
The FDA-approved drug Osimertinib binds with Mtb EF-Tu and inhibits *Mycobacterium* growth. **a**, **b** Thermal unfolding curves and unfolding transition midpoints of EF-Tu and EF-Tu/Osimertinib were detected by the Nano-DSF method. **c**–**f** Osimertinib did not affect *E. coli* growth, but significantly inhibited the growth of *M. smegmatis*, Mtb H37Ra, and *M. bovis* BCG strains. Different concentrations of Osimertinib were added to the bacterial strains, and cell densities were detected at different times (**c**–**e**). After treatment with varying concentrations of Osimertinib, the BCG strains were diluted 100 or 1000 times, and the numbers of colonies were calculated (**f**). **g** The MST result showed that Osimertinib is bound with EF-Tu in vitro. **h** The in silico modeling structure of EF-Tu and Osimertinib complex. EF-Tu was shown in a cartoon model. Osimertinib was shown in a stick model. The error bars represented the standard deviations (SD). *P < 0.05, **P < 0.01, ns no significance.

To identify the potential inhibitor of Mtb EF-Tu, we screened the FDA-approved drug library (total of 1971 drugs) with the nano-DSF method^40^. Compared to EF-Tu itself, sixteen drugs could dramatically affect the stability of the EF-Tu protein (Table 2). Significantly, Osimertinib, a medicine for treating non-small-cell lung carcinomas (NSCLC) with specific mutations^41^, significantly changed the unfolding transition midpoint from 52.5 to 60.4 °C (Fig. 6a, b). Next, 16 drugs were added to different bacterial strains to check the in vivo function separately. The results showed that Osimertinib did not change the growth of *E. coli*; however, it dramatically inhibited the growth of *M. smegmatis* and Mtb H37Ra strains at around 10 µM concentration (Fig. 6c–e). In contrast, other drugs did not show significant antibacterial activities, including hydroquinone and saractinib (Supplementary Fig. 4a, b). Also, Osimertinib significantly inhibited the growth of the *M. Bovis* BCG strain (Fig. 6f). The first-line anti-MTB drug isoniazid (also called INH) inhibits Mtb growth by disturbing the biosynthesis of mycolic acid. Consistently, isoniazid showed a strong ability to inhibit the growth of Mtb H37Ra and *M. smegmatis*, but did not change the growth of *E. coli* (Supplementary Fig. 4d–f). Thus, Osimertinib had a similar bacterial inhibition capacity with isoniazid in bacterial specificity. To further identify whether Osimertinib could directly bind with Mtb EF-Tu, we performed the microscale thermophoresis (MST) method with the purified protein. The result showed that Osimertinib bound to EF-Tu in vitro with medium intensity (Kd = 207 μM) (Fig. 6g), with the binding of GDP and EF-Tu as a positive control (Supplementary Fig. 4c).

**Table 2:** Drugs changed the thermal stability of Mtb EF-Tu.

Name	Target	Pathway	Tm (°C)
Doxazosin	Adrenergic receptor	Neuronal signaling	47
Fenoterol hydrobromide	Adrenergic receptor	Neuronal signaling	45.4
Metoprolol tartrate	Adrenergic receptor	Neuronal signaling	59.5
Domperidone	Dopamine receptor	Neuronal signaling	45.8
Alvimopan	Opioid receptor	Neuronal signaling	56.9
Motolimod	TLR	Immunology	48
Montelukast	LTR	Immunology	43.2
Estriol	Estrogen receptor	Endocrinology	44
Aliskiren temifumarate	RAAS	Endocrinology	46.8
Canagliflozin hemihydrate	SGLT	GPCR & G protein	44.1
Lixisenatide	Glucagon receptor	GPCR & G protein	55.4
Osimertinib (AZD9291)	EGFR	Protein tyrosine kinase	60.2
Saracatinib (AZD0530)	Src	Angiogenesis	61.1
Carvedilol Phosphate	Others	Others	75.9
Thioridazine hydrochloride	Others	Others	58
Aliskiren	Others	Others	46.7

We next screened thousands of crystallization conditions for solving the Osimertinib-bound EF-Tu complex structure; however, no qualified diffraction datasets were obtained. Thus, we built the in silico modeling complex structure of EF-Tu and Osimertinib using the AlphaFold2 software. The structure showed that Osimertinib bound with the Domain I and II of EF-Tu, composing of flexible loops and different from the site of the GDP-binding pocket (Fig. 6h). Also, the electrostatic potential surface of the osimertinib-binding pocket was full of negative charges (Supplementary Fig. 5a). Furthermore, the binding pocket of osimertinib in the human EGFR/osimertinib complex structure (PDB ID: 6LUD) is composed of flexible loops (Supplementary Fig. 5b). Next, we analyzed the binding sites of four known inhibitors in EF-Tu. The crystal structure of antibiotic enacyloxin/EF-Tu complex (PDB ID: 2BVN) shows that enacyloxin locates on the cleft between Domain I and III of *E. coli* EF-Tu (Supplementary Fig. 5c), whereas antibiotic GE2270A locates on Domain II and does not interact with Domain I and III of *E. coli* EF-Tu (PDB ID: 3U6K) (Supplementary Fig. 5d). In addition, kirromycin shares a similar location with enacyloxin (Supplementary Fig. 5e), while pulvomycin locates in the middle hole, which interacts with three domains of *T. ther* EF-Tu (Supplementary Fig. 5f), indicating that Osimertinib docking site may overlap partially with GE2270A and pulvomycin. Thus, Osimertinib might inhibit Mtb growth by blocking the rearrangement of different domains of EF-Tu.

## Discussion

Elongation factors play essential roles in synthesizing new proteins through translation in the ribosome. EF-Tu is one of the most critical proteins in bacteria, as it is the only protein delivering aminoacyl-tRNA to the ribosome for polypeptide synthesis^23,24^. Once EF-Tu exhibits dysfunction, the protein synthesis would be interrupted, and the growth of bacteria would be inhibited^42^. Unlike other bacteria, Mtb exhibits specific properties, including slow growth and latency, making it challenging to develop therapeutic targets^1^. Although the EF-Tu structures of *E. coli* and *T. thermophilus* have been solved for years, little is known about the Mtb EF-Tu. Our current work revealed the Mtb EF-Tu•EF-Ts complex structure (Fig. 1), and found that EF-Ts changed conformation upon binding with and reactivating EF-Tu (Figs. 2–4). Moreover, we identified the essential residues of EF-Ts, R13, N82, and H149, which affected the EF-Tu/EF-Ts complex formation using the SEC methods (Fig. 2). Although EF-Tu shared high sequence similarity and overall architecture, it bound to EF-Ts with different ratios between Mtb and *E. coli*. Most important, the structural difference existed in the C-terminal domain of *E. coli* EF-Ts, which stretched out and might block the interacting interface between EF-Ts and EF-Tu (Fig. 3).

Furthermore, our data revealed that the EF-Tu structure was retrieved back upon conformational change from the inactive form (EF-Tu•GDP) to the recycling form (EF-Tu/EF-Ts), explaining the recycling process of Mtb EF-Tu (Fig. 4). The GDP-binding pocket of EF-Tu was conserved across all GTPase proteins; however, the essential related residues dramatically shifted upon binding with EF-Ts, resulting in GDP dissociated from EF-Tu (Fig. 4b, e). This could clarify how EF-Tu as a G-protein was reactivated by EF-Ts during protein translation in Mtb.

EF-Tu is a promising target for exploring antibiotics^23^. Four kinds of antibiotics were designed to block the function of EF-Tu. Kirromycin binds with the 30S subunit of *E. coli* ribosome and interferes with the polypeptide synthesis^43^. Also, kirromycin significantly affects the nucleotide binding of unphosphorylated *Mtb* EF-Tu, but not the phosphorylated protein^44^. Pulvomycin can inhibit polypeptide synthesis in *B. brevi*s and *E. coli*^45^. In addition, enacyloxin Iia is active against different Gram-positive and Gram-negative bacteria except for Mtb and *M. Smegmatis*^46^. Antibiotic GE2270A can inhibit Gram-positive bacteria^47^. However, none was used for curing tuberculosis^48^. In this study, Osimertinib was identified to inhibit different Mycobacterium strains by directly targeting EF-Tu (Fig. 6). As a medicine for NSCLC with specific mutations, Osimertinib could potentially be used to treat TB, despite further investigations needing to be performed.

The structure prediction software, like AlphaFold2, is very powerful for providing the initial model. In this case, the AlphaFold2-predicted EF-Ts structure was successfully used as a search model to solve the Mtb EF-Tu•EF-Ts complex structure. When superimposed the two EF-Ts structures (PDB ID: 7VMX and P9WNM1-F1), we found that the core and N-terminal domains shared the same conformation, and the RMSD value was 0.627 Å (Supplementary Fig. 1c). As it was challenging to obtain the EF-Tu/osimertinib complex structure via X-ray crystallography, we built the in silico model structure by autodocking. Compared with other structures of EF-Tu and its inhibitors, Osimertinib is more likely located on the cleft between Domain I and II (Fig. 6 and Supplementary Fig. 5), whereas the mechanism needs to be explored with more evidence. Together, the detailed Mtb EF-Tu•EF-Ts complex and EF-Tu•GDP structural information provided an exquisite image for understanding the polypeptide elongation process of protein synthesis and might have practical implications for designing new medicine to cure tuberculosis.

## Methods

### Protein expression and purification

The full-length EF-Tu (Uniprot ID: P9WNN1) and EF-Ts (Uniprot ID: P9WNM1) were subcloned from the Mtb genomic library into the pSMT3 vector with a His-SUMO tag. The wild-type and mutants of EF-Tu and EF-Ts plasmids were transformed into *E. coli* BL21 (DE3) or Rosetta (DE3) cells for protein expression. *E. coli* cells were cultured in LB medium with 25 µg/ml kanamycin and 34 µg/ml chloramphenicol at 37 °C. Isopropyl β-D-1-thiogalactopyranoside (IPTG, 0.2 mM) was added to cells to induce protein expression at 20 °C for 18 h when the OD_600_ reached 0.6–0.8. Then, the cells were harvested by centrifugation at 6000 rpm for 15 min at 4 °C. The cell pellets were resuspended in a lysis buffer (Tris-HCl pH 8.0, 100 mM NaCl, 10 mM imidazole pH 8.0, 5% glycerol, 4 mM MgCl_2_, and 2 mM β-mercaptoethanol) and disrupted using a high-pressure homogenizer (JNBIO, China). The cell debris was removed by centrifugation at 17,000 rpm for 60 min at 4 °C. Next, the supernatant was purified using a His TrapTM HP column (GE Healthcare) and was eluted in a buffer (50 mM Tris-HCl, 500 mM NaCl, 250 mM imidazole, 5% glycerol, pH 8.0). The His-sumo tag was removed by the ULP1 enzyme cleavage, followed by additional Ni-NTA affinity chromatography as previously described^49^. The protein was further purified by gel filtration using a Superdex200 16/600 column (GE Healthcare) in a gel-filtration buffer (20 mM Tris-HCl pH 7.4, 100 mM NaCl, 2 mM DTT). The EF-Tu•EF-Ts complex was obtained by premixing the EF-Tu and EF-Ts proteins with a 1:1 molar ratio on ice for 30 min, and then the mixture was applied to a gel-filtration chromatography (Superdex200 16/600 GL).

### Mutagenesis

The EF-Ts mutants K24A, L21A, R13A, D77A, N82A, H149A, D154A, and EF-Tu-N358A were generated by whole-plasmid PCR in a 20-cycle reaction with steps at 98 °C for 10 s, 55 °C for 30 s, and 72 °C for 3 min per cycle. After digestion with the enzyme DpnI, the PCR products were transformed into *E. coli* Top10 cells. The positive constructs were determined by DNA sequencing.

### Dynamic light scattering (DLS) measurement

The DLS data were collected on the DYNAMICS software from DynaPro NanoStar (Wyatt Technology), operating at a light source wavelength of 658 nm and a fixed scattering angle of 90°. The fresh proteins were diluted to 1 mg/mL with a buffer containing 20 mM Tris-HCl (pH 8.0), 100 mM NaCl, 2 mM TCEP, and 5% glycerol at 25 °C.

### Crystallization and data collection

The EF-Tu•GDP and EF-Tu•EF-Ts complexes were crystallized using the sitting-drop vapor-diffusion method by mixing 0.2 μL protein and 0.2 μL reservoir solution at 18 °C. EF-Tu and GDP were premixed with a molar ratio of 1:5 on ice for 30 min before screening the crystallization conditions. EF-Tu•GDP crystals were grown in a reservoir solution containing 26% (w/v) PEG 6000 and 100 mM HEPES pH 7.0 with a protein concentration of 10 mg/mL. For the EF-Tu•EF-Ts complex, the two proteins were premixed with a molar ratio of 1:1 on ice for 30 min before screening. EF-Tu•EF-Ts crystals were grown in a reservoir solution containing 8% PEG 6000, 2 mM ZnCl_2_, and 0.1 M Tris-HCl pH 8.0 with a protein concentration of 5 mg/mL. All of the crystals were briefly soaked in a cryoprotectant solution consisting of 25% (v/v) glycerol dissolved in their corresponding mother liquors before being flash-cooled directly in a liquid-nitrogen stream at 100 K. The X-ray diffraction data were collected at the BL17U1 and BL19U1 beamlines of the Shanghai Synchrotron Radiation Facility. The process of data integration, scaling, and merging was performed using the HKL3000 package and the XDS program.

### Structure determination and refinement

The crystal structure of Mtb EF-Tu•GDP was determined by molecular replacement using the crystal structure of *E. coli* EF-Tu (PDB ID: 1EFC) as the search model. The EF-Tu•EF-Ts complex structure was determined by molecular replacement using EF-Tu•GDP and AlphaFold2-predicted EF-Ts structure (https://alphafold.ebi.ac.uk/entry/P9WNM1) as search models. Cycles of refinement and model building were carried out by using Phenix and COOT programs. The quality of the final model was evaluated with PROCHECK. All of the structures were displayed and analyzed using PyMOL program. The collected data and refinement statistics are summarized in Table 1.

### Model building of EF-Tu and Osimertinib complex

The model building process was described previoulsy^50^. In brief, the Mtb EF-Tu structure (PDB: 7VMX) and the simplified molecular-input line-entry system (SMILES) file of osimertinib were initially placed in the models. Optimized binding poses of Mtb EF-Tu/osimertinib complex were predicted by iteratively employing molecular docking using AutoDock Vina^51^. The 3D structure model was generated from the predicted distance and orientation using constrained minimization. Next, five best models of the top 50 folded structures satisfying the restraints were selected according to Rosetta energy and GOAP energy^51^. Then, the best complex model was selected and depicted by Pymol.

### Small angels X-ray scattering (SAXS)

The SAXS data of EF-Tu, EF-Ts, and EF-Tu•EF-Ts were collected at beamline BL19U2 of the Shanghai Synchrotron Radiation Facility, with a radiation wavelength of 1.03 Å. The protein samples were prepared at 0.1–10 mg/mL concentrations in the buffer (20 mM Tris-HCl pH 7.4, 100 mM NaCl). The samples were measured in triplicate, and the sample measurements were adjusted by subtracting the scattering from the buffer alone. Data were analyzed using the software package BioATSAS (https://www.embl-hamburg.de/biosaxs/). The scattering images were averaged and subtracted from the buffer-scattering images. Then, using the indirect Fourier transform method, the Rg was estimated. The distribution function p(r) was calculated from the parameter as Dmax. The SAXS envelopes of EF-Tu, EF-Ts, and EF-Tu•EF-Ts were built by GASBOR, as previously reported^33^.

### Isothermal titration calorimetry (ITC)

ITC assays were carried out on the MicroCal ITC200 calorimeter at 25 °C. The titration buffer for the proteins was 10 mM HEPES pH 8.0, 50 mM NaCl, and 2 mM TCEP. The protein concentrations were 0.1–1 mM. The fitting curve with a single-binding-site model was performed by the ITC data analysis module provided by the manufacturer.

### Nano differential scanning fluorimetry (Nano-DSF) experiments

Nano-DSF experiments were carried out on the Nano Temper Promethus NT48 instrument. The protein samples were prepared at 0.5 mg/mL concentration in the buffer (20 mM Tris-HCl pH 8.0, 100 mM NaCl, 2 mM TCEP, and 5% glycerol) after gel-filtration purification. Protein samples were transferred to capillary tubes after centrifugation (12,000 rpm, 20 °C, 10 min). The data were collected under the fluorescence absorption value within the range of 200–2000. The start temperature was 20 °C, and the highest temperature was 95 °C, with the heating rate being 1 °C/min to detect the T_m_ value of the protein. EF-Tu and Osimertinib were mixed in a molar ratio of 1:3 at room temperature for 20 min with the same data collection process for EF-Tu.

### MicroScale thermophoresis (MST) experiments

The purified EF-Tu was dialyzed into the SEC buffer and labeled by the cyanine 5-NHS ester iodide (MCE, HY-135414B) according to the protocol. Osimertinib (Selleck, S7297) was dissolved in DMSO at 10 mM. The MST experiments were carried out on the Nano Temper Monolith NT.115 instrument. Labeled EF-Tu (200 nM) was mixed with the indicated concentrations of Osimertinib in the SEC buffer. The MST data were collected under 20% light-emitting diode power and 40% infrared laser power. The K_d_ value was determined by the Nano Temper analysis software (v.1.5.41).

### Bacterial culture

Mtb H73Ra (ATCC 25177), *M. Smegmatis* (ATCC 700084), and *M. Bovis* BCG (ATCC 35733) strains were cultured in the Middlebrook 7H9 medium with an extra 10% oleic acid-albumin-dextrose-catalase-enriched Middlebrook (OADC). All the bacteria were cultured at 37 °C.

### Antimicrobial activity assay

The Mtb H37Ra (ATCC 25177) and *M. Smegmatis* (ATCC 70084) strains were cultured to the logarithmic stage and transferred into a 96-well plate at a 10% inoculation rate, with 100 µL fresh Middlebrook 7H9 medium and 10% OADC per well. Strains were mixed with the indicated concentrations of Osimertinib in a 7H9 medium. The antimicrobial activity assay was carried out on the Spectra M5 plate reader (Molecular Devices) instrument. The absorption value of 600 nm (OD600) was collected every 30 min at 37 °C. *M. Bovis* BCG Danish strain (ATCC 35733) was grown in Middlebrook 7H9 broth and on Middlebrook 7H10 agar supplemented with 10% OADC at 37 °C. After three days of incubation of *M. Bovis* BCG with Osimertinib, the culture was diluted and cultured in 7H10 agar plates at 37 °C for 3–4 more weeks. Then, the CFU of bacteria was counted.

### Statistics and reproducibility

Each experiment was performed at least three times (*n* = 3). All experiment data were analyzed using GraphPad Prism 8.0 (GraphPad Software Inc., USA) and were presented as the mean ± SD. Statistical analysis was performed using Student’s *t* test or two-way ANOVA. A value of *P* < 0.05 was considered statistically significant.

### Reporting summary

Further information on research design is available in the Nature Research Reporting Summary linked to this article.

## Supplementary information

Supplementary Material

Description of Additional Supplementary Files

Supplementary Data 1

Supplementary Data 2

Reporting Summary

## Data Availability

The coordinates and structural factors have been deposited in the Protein Data Bank with accession codes 7VMX (EF-Tu•EF-Ts complex) and 7VOK (EF-Tu•GDP complex). The SAXS data and models have been deposited in the SASBDB database with accession codes SASDQB5 (EF-Tu), SASDQC5 (EF-Ts), and SASDQD5 (EF-Tu/EF-Ts complex). All data generated or analyzed during this study are included in this published article (and its supplementary information files). The source data for all plots are shown in Supplementary Data 1. The uncropped and unedited gel images are included in Supplementary Data 2. All other data are available from the corresponding author upon reasonable request.
