# Supplementary material for: Structural insights of the elongation factor EF-Tu complexes in protein translation of Mycobacterium tuberculosis
Source: Commun Biol. 2022 Oct 3;5:1052. doi: 10.1038/s42003-022-04019-y (PMC9529903; doi:10.1038/s42003-022-04019-y)
Supplement: Supplementary file 1 — Supplementary Material [file 42003_2022_4019_MOESM1_ESM.pdf]

Supplementary information

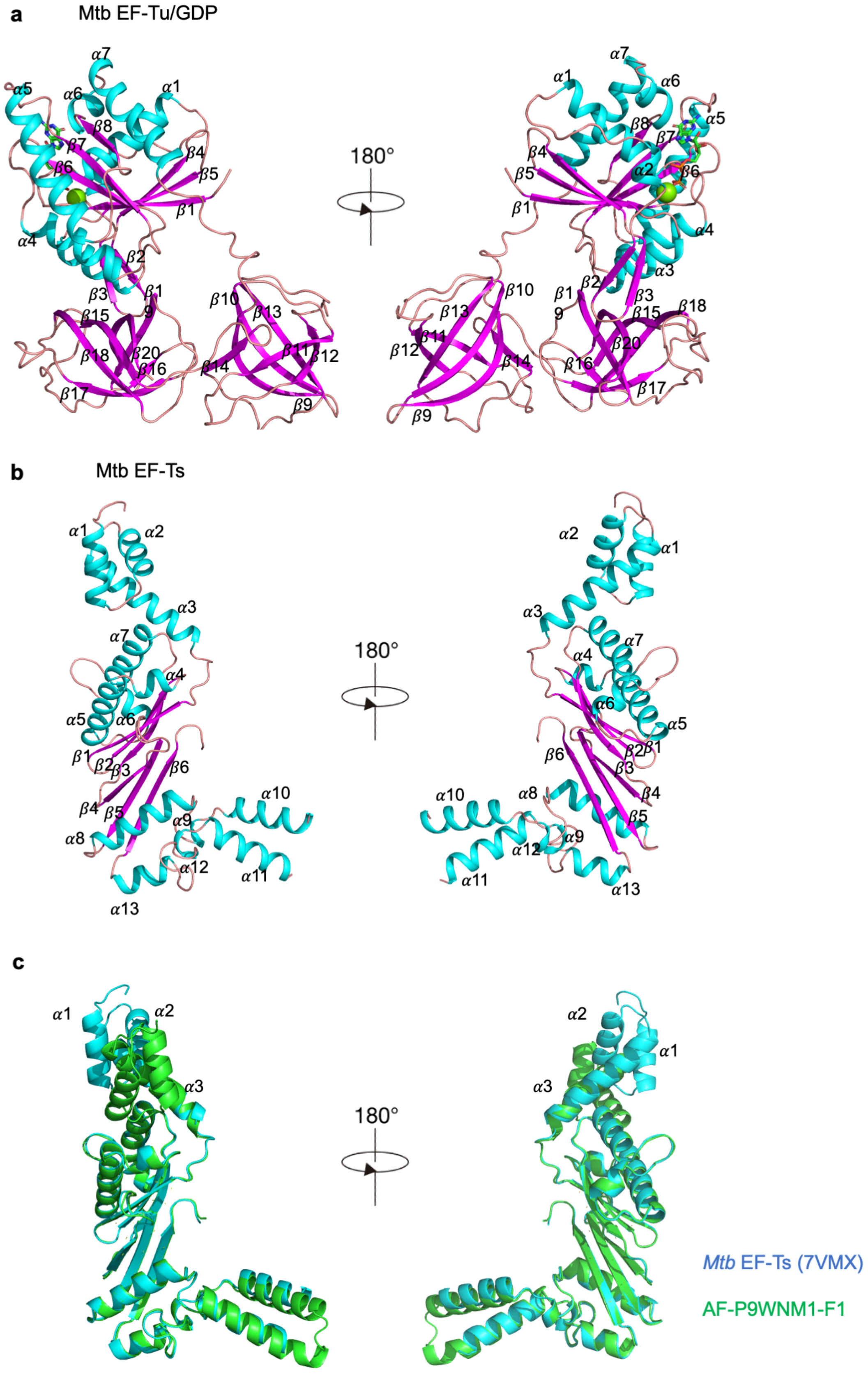

Supplementary Figure 1. The ribbon representations of the Mtb EF-Tu•GDP (PDB ID: 7VOK) (a) and EF-Ts (PDB ID: 7VMX) (b) structures. Structural superimposition of Mtb EF-Ts (cyan) and the AlphaFold predicted EF-Ts (green) was shown in **c**. The difference between the two structures is majorly located in the α1-α3 helices. The Mtb EF-Tu•GDP and EF-Ts were shown in cartoon models. α-helix and β-strand were colored with cyan and purple, respectively. The GDP molecule was shown in a stick model. Mg^{2+} was shown as a green sphere.

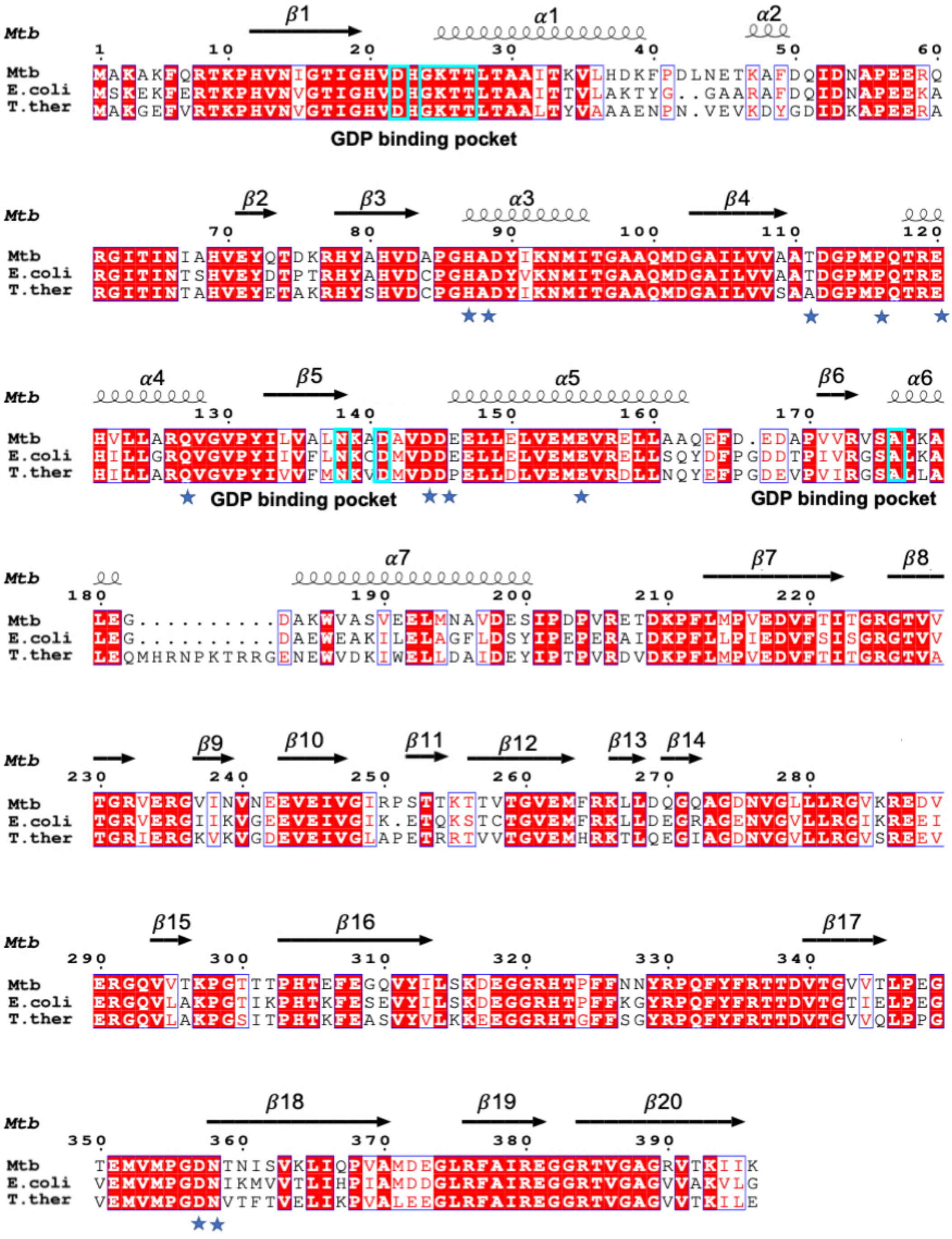

Supplementary Figure 2. Structure-based multiple sequence alignment of EF-Tu proteins among Mtb (PDB ID: 7VMX), *E. coli* (PDB ID: 1EFU), and *T. thermophilus* (PDB ID: 1AIP). Identical and similar residues among groups were shown in white text on a red background and red text on a white background, respectively. The second structure elements, including α-helix and β-strand, were

denoted as α and β, respectively. The residues related to the interface between EF-Tu and EF-Ts were labeled with blue asterisks. The GDP-binding pocket-related residues were labeled with cyan rectangles.

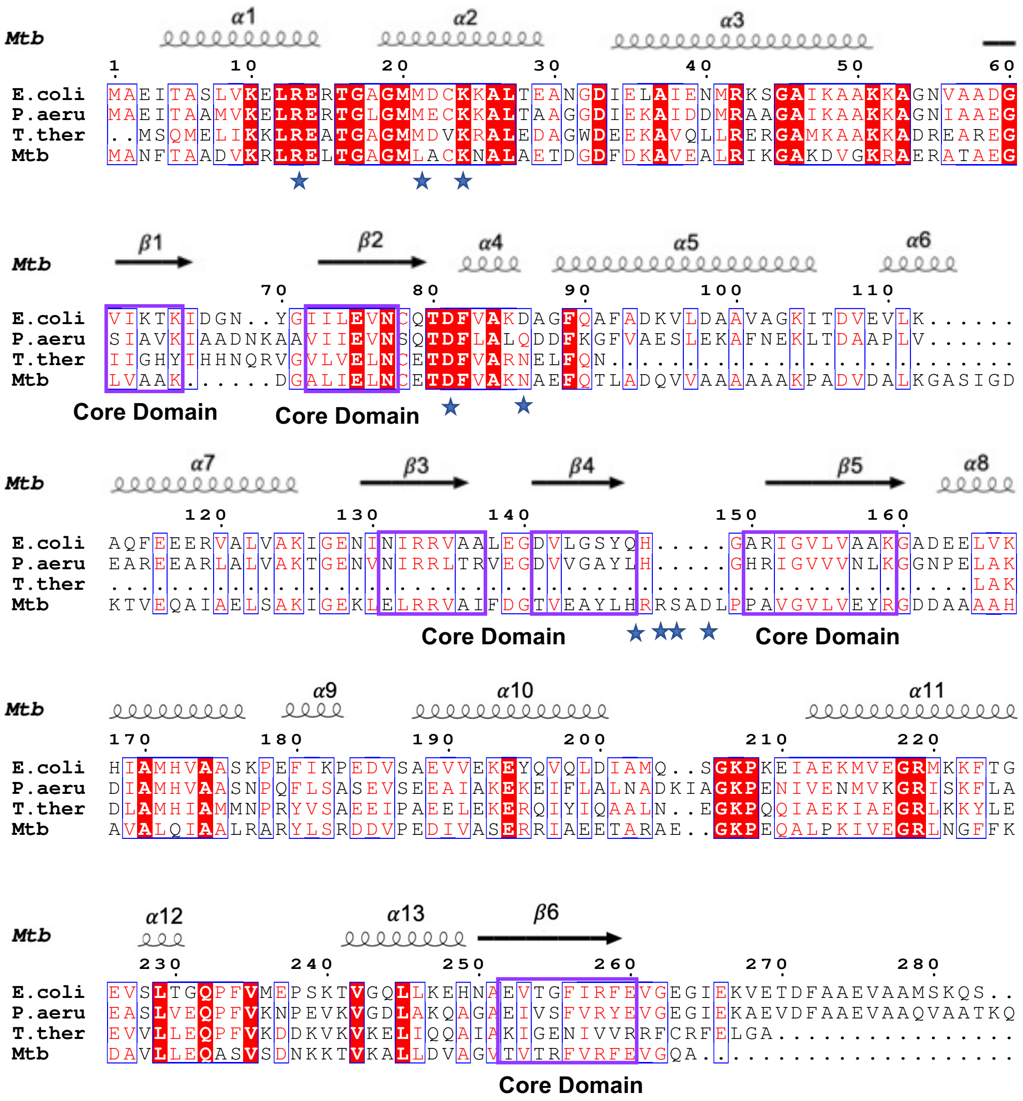

Supplementary Figure 3. Structure-based multiple sequence alignment of EF-Ts protein among Mtb (PDB ID: 7VMX), *E. coli* (PDB ID: 1EFU), *T. thermophilus* (PDB ID: 1AIP), and *P. aeruginosa* (AF-P9WNM1-F1). Identical and similar residues among groups were shown in white text on a red background and red text on a white background, respectively. The second structure elements, including α-helix and β-strand, were denoted as α and β, respectively. The residues related to the interface between EF-Tu and EF-Ts were labeled with blue asterisks. The critical residues of the core Domain were labeled with purple rectangles.

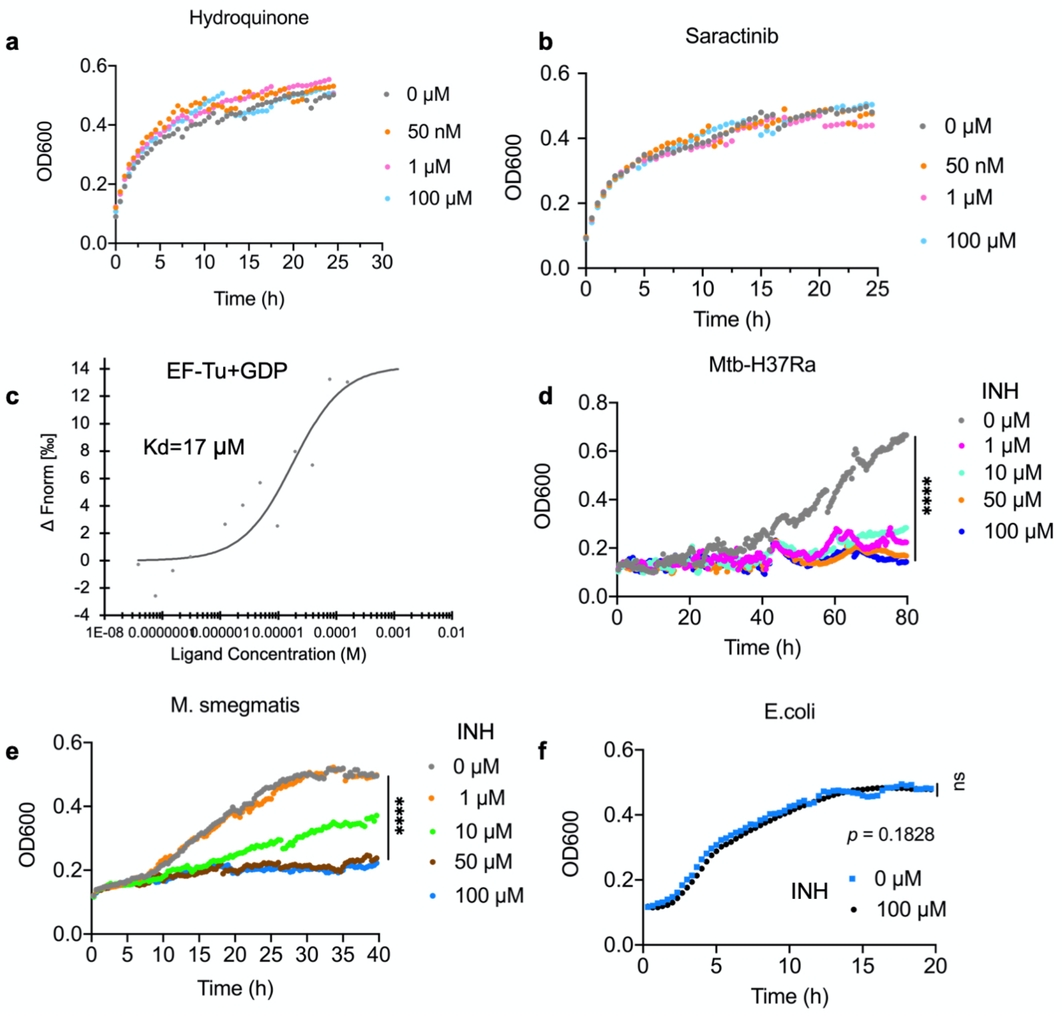

Supplementary Figure 4. Isoniazid (INH) exhibited anti-bacterial activity. a-b Hydroquinone and saractinib did not affect the growth of Mtb H37Ra. **c** The MST result showed that GDP bound with Mtb EF-Tu *in vitro*. **d-f** Isoniazid did not affect the growth of *E. coli,* but significantly inhibited the growth of Mtb H37Ra and *M. smegmatis*. Each experiment was repeated three times. The error bars represented the standard deviations (SD). * $p < 0.05$, ** $p < 0.01$, ns, no significance.

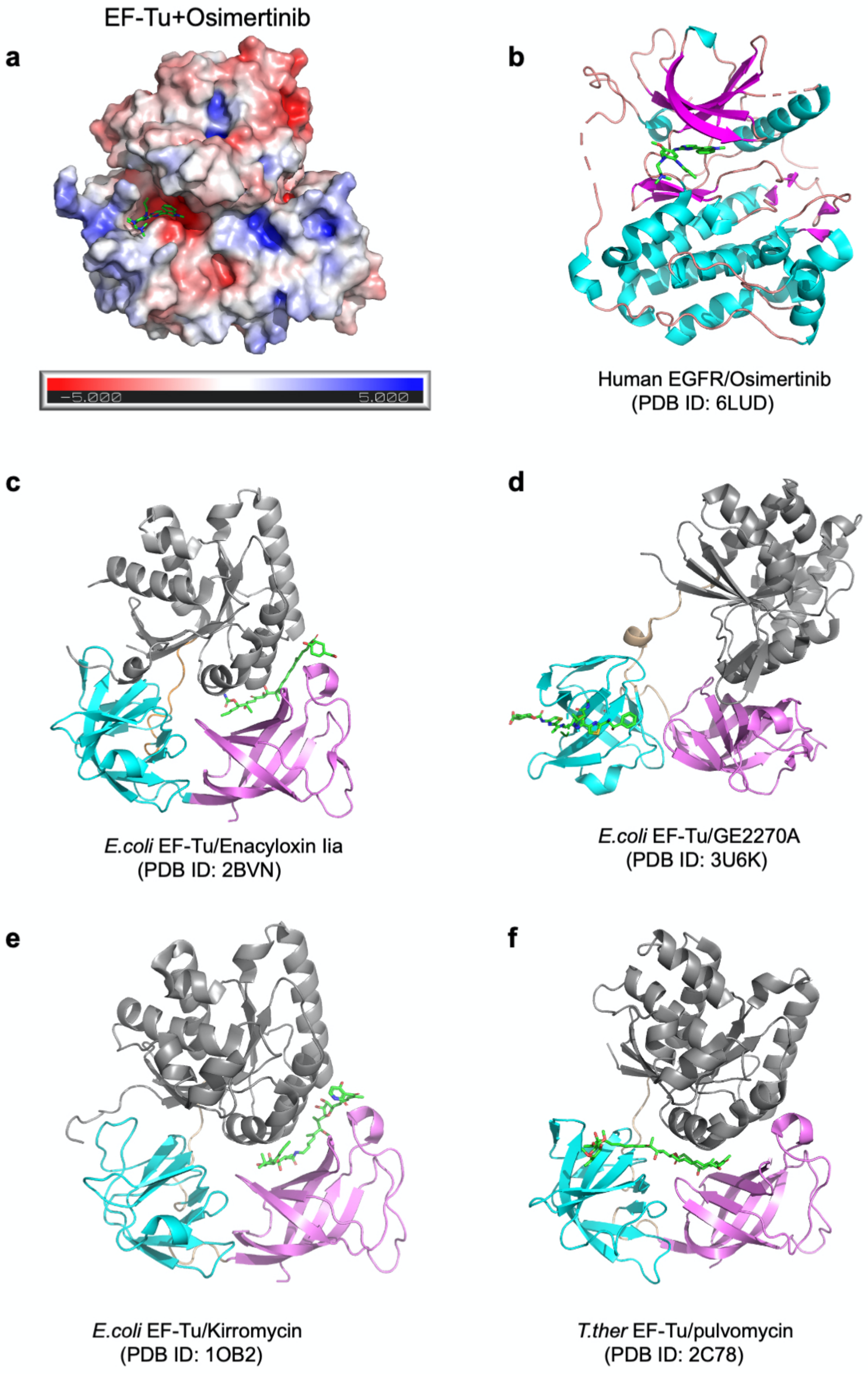

Supplementary Figure 5. Structural representations of ET-Tu with different inhibitors. a The electrostatic potential surface of the modeled EF-Tu•Osimertinib

complex. Red indicated the negative potential, and blue indicated the positive potential. Osimertinib was shown as sticks. **b** A ribbon representation of human EGFR•Osimertinib complex structure (PDB ID: 6LUD). Osimertinib was shown as sticks. **c-f** Ribbon representations of different EF-Tu/inhibitor complex structures. Enacyloxin Iia is located on the cleft between Domain I and III of *E. coli* EF-Tu (PDB ID: 2BVN) **c**. GE2270A binds with Domain II of *E. coli* EF-Tu (PDB ID: 3U6K) **d**. Kirromycin is located on the cleft between Domain I and III of *E. coli* EF-Tu (PDB ID: 1OB2) **e**. Pulvomycin is located in the hole between three domains of *T. ther* EF-Tu (PDB ID: 2C78) **f**. The color is shown as in Figure **1c**. The inhibitors were shown as sticks.
