# Supplementary material for: Structural insights of the elongation factor EF-Tu complexes in protein translation of Mycobacterium tuberculosis
Source: Commun Biol. 2022 Oct 3;5:1052. doi: 10.1038/s42003-022-04019-y (PMC9529903; doi:10.1038/s42003-022-04019-y)
Supplement: Supplementary file 2 — Description of Additional Supplementary Files [file 42003_2022_4019_MOESM2_ESM.pdf]

Description of Additional Supplementary Files

File name: Supplementary Data 1
Description: The source data for all plots.

File name: Supplementary Data 2
Description: The uncropped and unedited gel images.
