# Supplementary material for: Structural insights of the elongation factor EF-Tu complexes in protein translation of Mycobacterium tuberculosis
Source: Commun Biol. 2022 Oct 3;5:1052. doi: 10.1038/s42003-022-04019-y (PMC9529903; doi:10.1038/s42003-022-04019-y)
Supplement: Supplementary file 4 — Supplementary Data 2 [file 42003_2022_4019_MOESM4_ESM.pdf]

Supplementary information (unedited images)

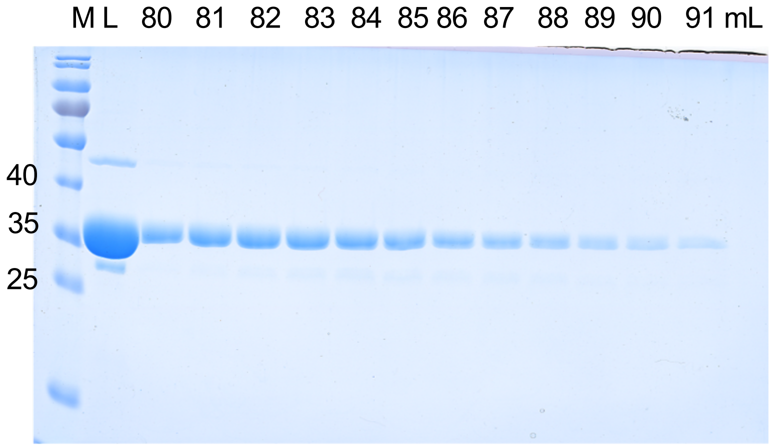

Figure 1b. The SDS-PAGE result of EF-Ts protein.

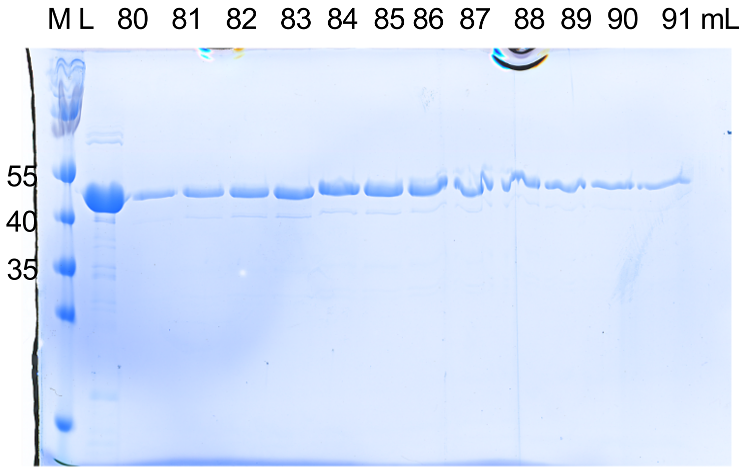

Figure 1b. The SDS-PAGE result of EF-Tu protein.

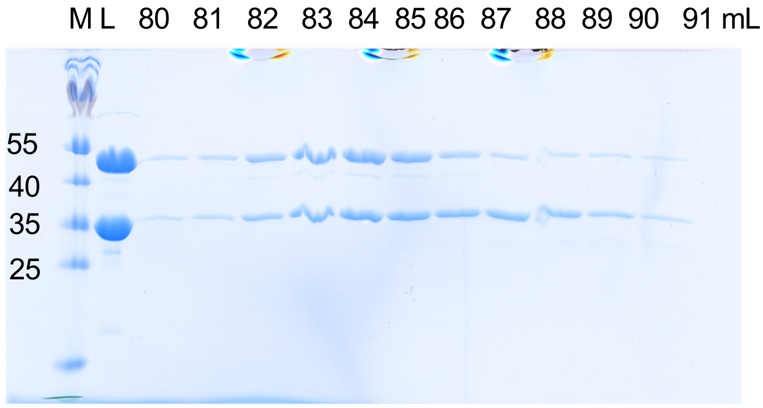

Figure 1b. The SDS-PAGE result of EF-Tu/EF-Ts complex.

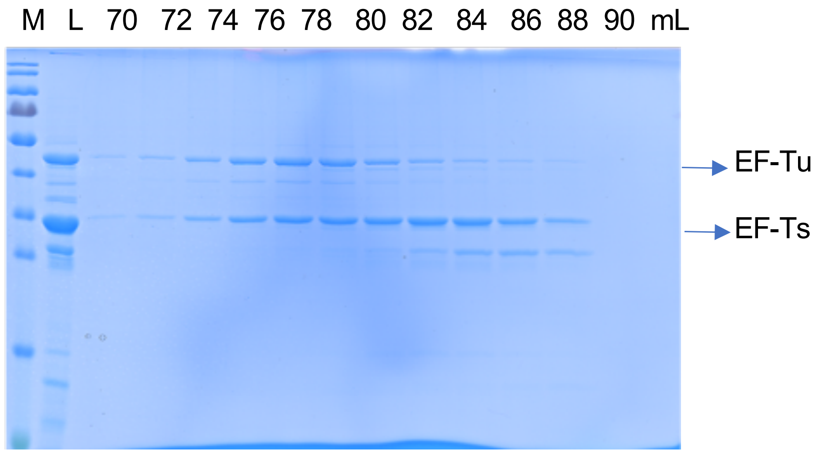

Figure 2d. The SDS-PAGE result of EF-Tu-N358A/EF-Ts complex.

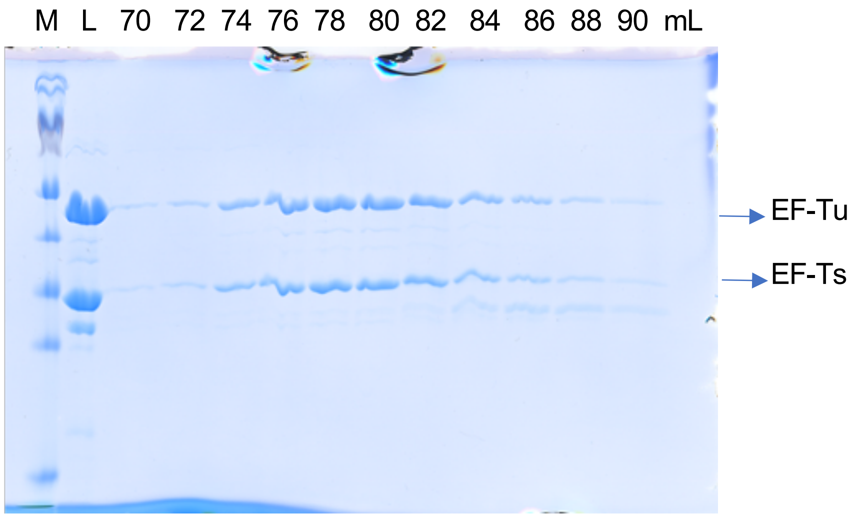

Figure 2d. The SDS-PAGE result of EF-Tu/EF-Ts-D77A complex.

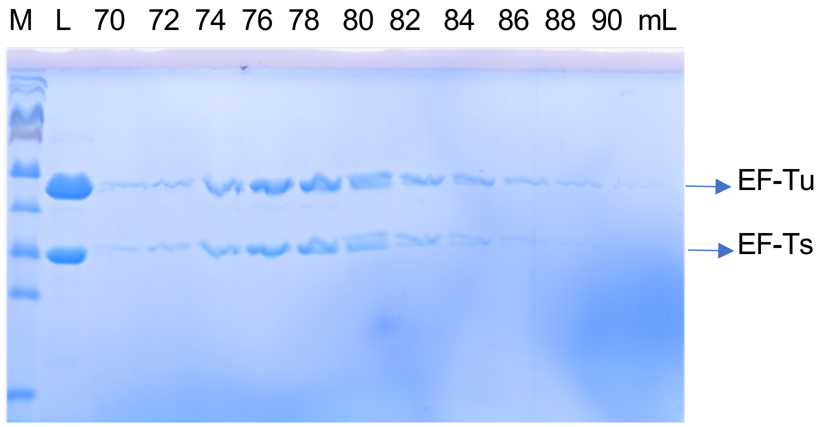

Figure 2d. The SDS-PAGE result of EF-Tu/EF-Ts-D154A complex.

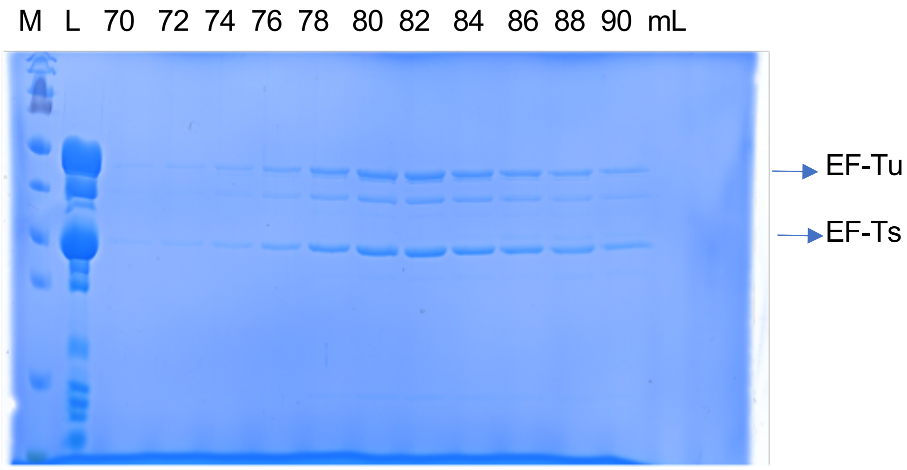

Figure 2d. The SDS-PAGE result of EF-Tu/EF-Ts-H82A complex.

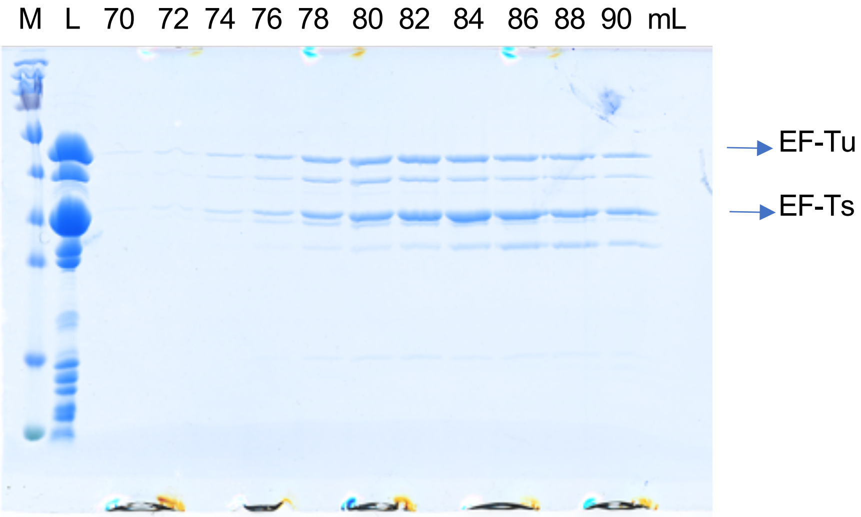

Figure 2d. The SDS-PAGE result of EF-Tu/EF-Ts-H149A complex.

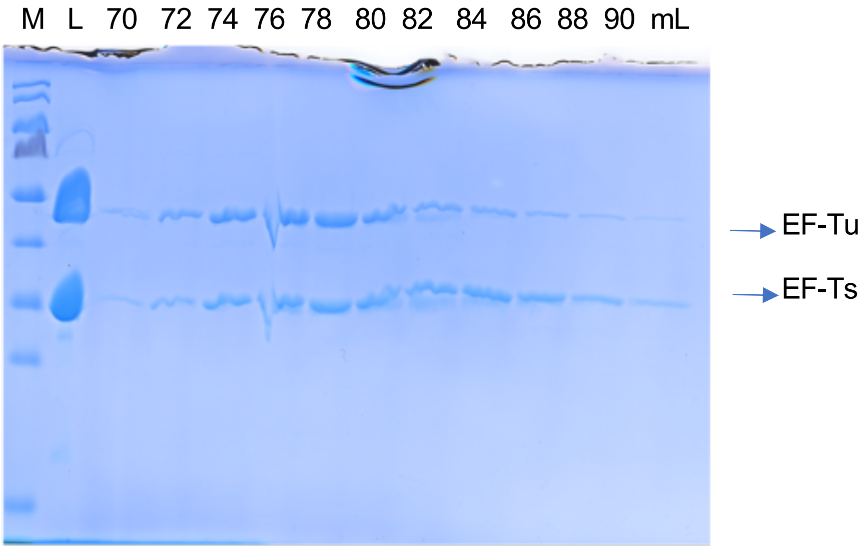

Figure 2d. The SDS-PAGE result of EF-Tu/EF-Ts-K24A complex.

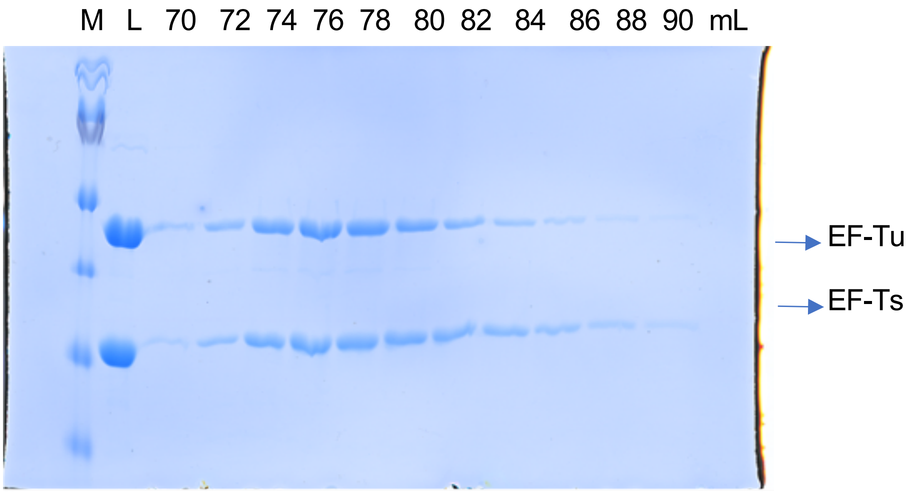

Figure 2d. The SDS-PAGE result of EF-Tu/EF-Ts-L21A complex.

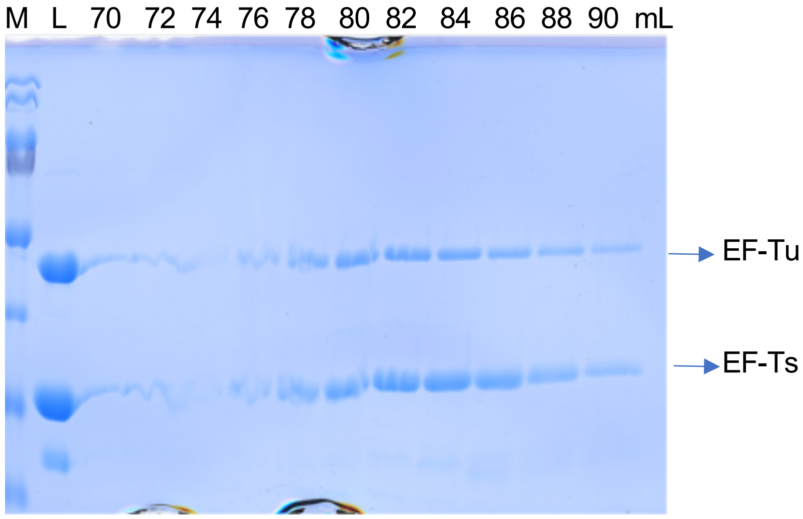

Figure 2d. The SDS-PAGE result of EF-Tu/EF-Ts-R13A complex.

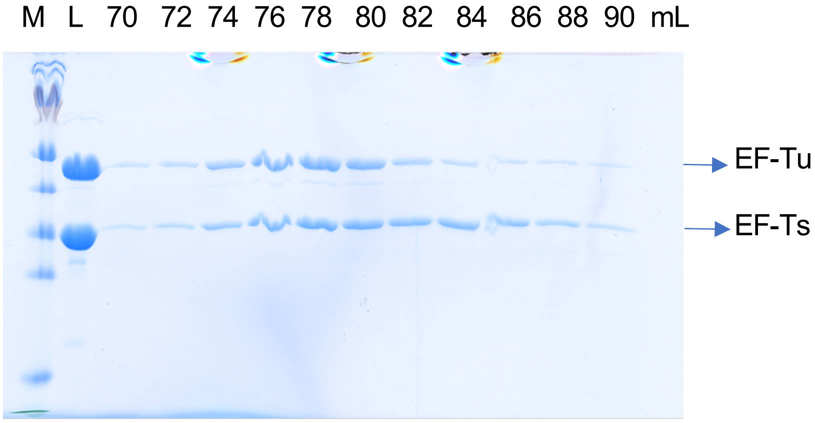

Figure 2d. The SDS-PAGE result of EF-Tu/EF-Ts complex.
