# Supplementary material for: Structural insights of the elongation factor EF-Tu complexes in protein translation of Mycobacterium tuberculosis
Source: Commun Biol. 2022 Oct 3;5:1052. doi: 10.1038/s42003-022-04019-y (PMC9529903; doi:10.1038/s42003-022-04019-y)
Supplement: Supplementary file 5 — Reporting Summary [file 42003_2022_4019_MOESM5_ESM.pdf]

nature portfolio

Corresponding author(s): Jixi Li, Guoping Zhao

Last updated by author(s): Sep 8, 2022

Reporting Summary

Nature Portfolio wishes to improve the reproducibility of the work that we publish. This form provides structure for consistency and transparency in reporting. For further information on Nature Portfolio policies, see our Editorial Policies and the Editorial Policy Checklist.

Statistics

For all statistical analyses, confirm that the following items are present in the figure legend, table legend, main text, or Methods section.

n/a	Confirmed	
☒	☐	The exact sample size (*n*) for each experimental group/condition, given as a discrete number and unit of measurement
☒	☐	A statement on whether measurements were taken from distinct samples or whether the same sample was measured repeatedly
☒	☐	The statistical test(s) used AND whether they are one- or two-sided<br>*Only common tests should be described solely by name; describe more complex techniques in the Methods section.*
☒	☐	A description of all covariates tested
☒	☐	A description of any assumptions or corrections, such as tests of normality and adjustment for multiple comparisons
☒	☐	A full description of the statistical parameters including central tendency (e.g. means) or other basic estimates (e.g. regression coefficient) AND variation (e.g. standard deviation) or associated estimates of uncertainty (e.g. confidence intervals)
☐	☒	For null hypothesis testing, the test statistic (e.g. *F*, *t*, *r*) with confidence intervals, effect sizes, degrees of freedom and *P* value noted<br>*Give P values as exact values whenever suitable.*
☒	☐	For Bayesian analysis, information on the choice of priors and Markov chain Monte Carlo settings
☒	☐	For hierarchical and complex designs, identification of the appropriate level for tests and full reporting of outcomes
☒	☐	Estimates of effect sizes (e.g. Cohen's *d*, Pearson's *r*), indicating how they were calculated

Our web collection on statistics for biologists contains articles on many of the points above.

Software and code

Policy information about availability of computer code

Data collection	X ray data collection: SSRF BEAMLINE BL19U1 (DECTRIS PILATUS3 6M)<br>SAXS data collection: SSRF BEAMLINE 19U2 (PILATUS 1M detector, DECTRIS, Switzerland)<br>ITC data collection: MicroCal ITC200<br>MST data collection: Monolith NY.115 instrument<br>nano DSF data collection: Promethus NT.48 instrument
Data analysis	X ray data analysis: CCP4 (version 7.1.015), PHENIX (version 1.18.1), Coot (version 0.9.5)<br>Sequence alignment: ClustalW and ENDscript<br>Molecular Graphics: Pymol (version 2.4.0)<br>SAXS data analysis: Primus (version 2.8.1)<br>Data plotting: GraphPad Prism (version 9.0)

For manuscripts utilizing custom algorithms or software that are central to the research but not yet described in published literature, software must be made available to editors and reviewers. We strongly encourage code deposition in a community repository (e.g. GitHub). See the Nature Portfolio guidelines for submitting code & software for further information.

Data

Policy information about availability of data

All manuscripts must include a data availability statement. This statement should provide the following information, where applicable:

- Accession codes, unique identifiers, or web links for publicly available datasets
- A description of any restrictions on data availability
- For clinical datasets or third party data, please ensure that the statement adheres to our policy

Atomic coordinates of the EF-Tu and GDP complex structure have been deposited in the Protein Data Bank (PDB) under accession number 7VOK. Atomic coordinates of the EF-Tu and EF-Ts complex structure have been deposited in the Protein Data Bank (PDB) under accession number 7VMX.

Human research participants

Policy information about studies involving human research participants and Sex and Gender in Research.

Reporting on sex and gender	There are no human research.
Population characteristics	There are no human research.
Recruitment	There are no human research.
Ethics oversight	There are no human research.

Note that full information on the approval of the study protocol must also be provided in the manuscript.

Field-specific reporting

Please select the one below that is the best fit for your research. If you are not sure, read the appropriate sections before making your selection.

☒ Life sciences ☐ Behavioural & social sciences ☐ Ecological, evolutionary & environmental sciences

For a reference copy of the document with all sections, see nature.com/documents/nr-reporting-summary-flat.pdf

Life sciences study design

All studies must disclose on these points even when the disclosure is negative.

Sample size	Sample sizes were not pre-determined and there are no sample size calculation was performed. No information was derived about a population based on sampling.
Data exclusions	In ITC and MST experiments, we discarded disordered points that could not be used in data fitting. This is a widely used and accepted practice in such analysis experiments. And no data were excluded from analysis.
Replication	The bacterial inhibiting experiments were performed three times and the results of MST were averaged over the three trials.
Randomization	This is not relevant to my study as there are no sample distribution.
Blinding	This is no relevant to my research as there are no experiments relating group allocation in macromolecular structure research. Each experiment was analyzed using consistent methods.

Reporting for specific materials, systems and methods

We require information from authors about some types of materials, experimental systems and methods used in many studies. Here, indicate whether each material, system or method listed is relevant to your study. If you are not sure if a list item applies to your research, read the appropriate section before selecting a response.

Materials & experimental systems

n/a	Involved in the study
☒	☐ Antibodies
☒	☐ Eukaryotic cell lines
☒	☐ Palaeontology and archaeology
☒	☐ Animals and other organisms
☒	☐ Clinical data
☒	☐ Dual use research of concern

Methods

n/a	Involved in the study
☒	☐ ChIP-seq
☒	☐ Flow cytometry
☒	☐ MRI-based neuroimaging
